# PITX1 suppresses osteosarcoma metastasis through exosomal LINC00662-mediated M2 macrophage polarization

**DOI:** 10.1007/s10585-022-10192-5

**Published:** 2022-11-05

**Authors:** Ying Zhang, Yelong Chen, Chuangzhen Chen, Huancheng Guo, Chunbin Zhou, Hu Wang, Zhaoyong Liu

**Affiliations:** 1 Department of Radiotherapy, Cancer Hospital of Shantou University Medical College, No. 7 Raoping Road, Shantou, 515041 Guangdong China; 2 Department of Orthopaedics, First Affiliated Hospital of Shantou University Medical College, No.57 Changping Road, Shantou, 515041 Guangdong China

**Keywords:** PITX1, Osteosarcoma, LINC00662, M2 macrophages, CCL22

## Abstract

Paired-like homeodomain transcription factor 1 (PITX1) is frequently downregulated in cancers, including osteosarcoma (OS). However, its role in OS remains unknown. Therefore, we aimed to explore the functions and potential mechanisms of PITX1 in OS malignant progression. Elevated PITX1 suppressed OS cell proliferation and migration, based on transwell, proliferation, and colony formation assays. Pathway enrichment analysis of differentially-expressed genes between PITX1-overexpressing and control OS cells indicated that PITX1 expression was associated with the FAK/Src and PI3k/Akt signaling pathways. Mechanistically, ubiquitination assays and rescue experiments showed that PITX1 interacted with transcription factor STAT3, leading to decreased STAT3 transcriptional activity, which repressed the expression of LINC00662. Specific knockdown of LINC00662 reduced the tumor growth and invasion of OS cells induced by downregulated PITX1. Moreover, exosomal LINC00662, derived from PITX1 knockdown OS cell lines activated M2 macrophages in cell co-culture assays. M2 macrophage secreted several cytokines, among which CCL22 was found to cause OS cell EMT. Collectively, our data indicate that PITX1 suppresses OS cell proliferation and metastasis by downregulating LINC00662. Moreover, LINC00662 can be packaged into OS cell-derived exosomes to mediate M2 macrophage polarization to promote OS metastasis via CCL22.

**Supplementary Information:**

The online version contains supplementary material available at 10.1007/s10585-022-10192-5.

## Introduction

Osteosarcoma (OS) is one of the most common primary malignant bone tumors in children. The 5-year survival has remained at 60–70% in OS patients, but among patients with lung metastases, the 5-year survival is only 20% [1]. Therefore, identification of new prognostic markers and potential mechanisms of OS metastasis are of importance for its treatment and prognosis.

Paired-like homeodomain transcription factor 1 (PITX1), located on human chromosome 5q31, is a bicoid-related transcription factor for the pro-opiomelanocortin gene, and is involved in both pituitary cell differentiation and hind limb development [2]. PITX1 is often downregulated in cancer compared with normal tissues and is positively associated with patient survival in lung cancer, gastric cancer, breast cancer and esophageal cancer [3–6]. Thus, PITX1 is considered a potential tumor suppressor. In melanoma cell lines, the molecular mechanisms underlying how PITX1 suppresses carcinogenesis have been shown to involve inhibiting RAS activity and TERT expression [7]. Previously, we showed that PITX1 is down-regulated in OS tissues and its expression is negatively correlated with lung metastasis [8]. However, the detailed molecular mechanisms underlying how PITX1 represses OS development and progression remain unclear.

LncRNAs, a class of noncoding RNAs longer than 200 nucleotides in length, regulate the transcription, stability and translation of protein-coding genes, and participate in important biological processes related to human cancer [9]. Intriguingly, lncRNAs are also packaged into exosomes and involved in cell–cell communication in the tumor microenvironment (TME). Such exo-lncRNAs have been implicated in the development, proliferation, invasion, angiogenesis, and drug resistance of tumors [10]. The *LINC00662* gene, located on chromosome 19, encodes a 2085 bp lncRNA that is highly expressed in cancer, such as breast cancer and hepatocellular carcinoma [11, 12]. Mechanistically, LINC00662 promotes tumor progression via interacting with both proteins and RNAs, as well as via regulating the Wnt/β-catenin, SMD and Hippo signaling pathways [13]. These findings indicate that LINC00662 may be a potential biomarker for cancer diagnosis and prognosis.

Tumor-associated macrophages (TAMs) constitute a cellular subset of the tumor microenvironment and are involved in the growth and progression in various cancers, including OS [14, 15]. In OS, there is a higher density of M2-type TAMs, and TM-related pro-inflammatory molecules, in lung metastases compared with primary OS tumors, indicating a role for M2-type TAMs in OS invasion [16]. Several studies demonstrate that TAMs can activate intracellular signaling pathways to promote angiogenesis in OS [17, 18]. Furthermore, blocking M2-polarization can reduce expression of stem cell-like properties and inhibit OS metastasis [14]. However, the mechanisms underlying the activation of M2 macrophages by tumor cells and how M2 macrophages affect tumor progression remain unclear.

In this study, we determined the function of PITX1 in human OS cells and showed that PITX1 inhibits OS cell migration, proliferation, and epithelial-mesenchymal transition (EMT) via suppression of LINC00662. Furthermore, LINC00662 stimulated M2 macrophage polarization, which in turn enhanced OS cell migration via the C–C motif chemokine 22 (CCL22). These findings delineate the tumor suppressor role of PITX1 in OS development and suggest PITX1 may be a potential therapeutic target for OS.

## Materials and methods

### Patient samples and cell lines

OS tumor and normal bone samples were collected from Sun Yat-sen University Cancer Center and the First Affiliated Hospital of Shantou University Medical College. None of the patients received preoperative radiotherapy or chemotherapy before the surgery and all patients were provided written informed consent. This study was approved by the ethical review committees of the Shantou University Medical College Cancer Center.

OS cell lines (U2OS, HOS, Saos-2 and MG63) were purchased from the National Collection of Authenticated Cell Cultures and maintained in Dulbecco’s modified Eagle’s medium (DMEM) (Gibco, Waltham, MA, USA) supplemented with 10% fetal bovine serum (FBS, Gibco) and a streptomycin, penicillin mixture (Gibco). THP-1 monocytes were differentiated into M0 macrophages by incubation with 100 nm phorbol 12-myristate 13-acetate (PMA) (Sigma-Aldrich, St Louis, MO, USA) for 24 h. Differentiated M0 macrophages were treated with 40 ng/ml IL-4 (PeproTech, New Jersey, USA) to further differentiate into M2 macrophages. All cell lines were authenticated by short tandem repeat (STR) profiling and confirmed to be mycoplasma negative.

### Immunohistochemistry (IHC) and immunofluorescence (IF)

All tumor or mouse tissues were fixed in 10% formalin and embedded in paraffin. For immunohistochemical (IHC) staining of tissue sections, an IHC staining kit (MXB biotechnologies, Fujian, China) was used according to the manufacturer’s instructions. PITX1 (1:50, ab244308, Abcam, USA) and CD163 (1:200, Cell Signaling Technology, USA) were used as primary antibody. Paraffin-embedded sections or cells were used to conduct IF staining as previously described [19]. Proteins were detected with antibodies against E-cadherin (#14472,1:400), vimentin (#5741,1:400), β-catenin (#8480,1:400), p-β-catenin (#4176,1:400), purchased from Cell Signaling Technology (CST). The seconday antibodies were Anti-Mouse or Rabbit IgG H&L (Alexa Fluor® 647) (ab150115, ab150075) and Anti- Mouse or Rabbit IgG H&L (Alexa Fluor® 488) (ab150113, ab150077) purchased from Abcam.

### Drug, siRNAs, plasmid constructs, transfection and stable cell lines

The proteasome inhibitor Z-Leu-Leu-Leu-al (MG132) was purchased from MCE (Shanghai, China), the protein synthesis inhibitor cycloheximide (CHX) was purchased from Sigma (USA), and siRNAs targeting PITX1 and STAT3 were purchased from GenePharma (Jiangsu, China). Plasmids expressing Flag-tagged PITX1 and Myc-tagged wildtype or domaindeleted STAT3, as well as empty vectors (NC) were purchased from the Vigene (Shandong, China). For transfection, 1 × 10^6^ OS cells were inoculated into each well of a 6-well culture dish and transiently transfected with 2 μg plasmid using Lipofectamine 3000 reagent (Invitrogen) as per the manufacturer’s suggested protocol. For stable cell lines, PITX1, short hairpin (sh)-LINC00662 or LINC00662 was cloned into a lentiviral expression vector with fluorescent tags GFP from Vigene. The packaged lentivirus was used to infect OS cells following the manufacturer’s instructions.

### Protein extraction and western blotting

Cells were lysed in RIPA buffer (Beyotime, Jiangsu, China) containing a protease inhibitor cocktail, and the cell extracts were subjected to western blotting as previously described [19]. Primary antibodies were against PITX1 (1:500, Abcam, USA), cleaved-caspase 3, caspase 3, PARP, cleaved-PARP, FAK, p-FAK, Src, p-Src, Akt, p-Akt, PI3K, p-PI3K, E-cadherin, vimentin, β-catenin, p- β-catenin, Myc, TGS101, CD63, CD81, CD163, Flag, STAT3, ubiquitin, GAPDH and β-actin (1:1000, Cell Signaling Technology, USA), as well as CCL22 (1:1000, Abnova, Guangzhou, China). The secondary antibodies used were anti-Rabbit (CST, #7074, 1:1000) and anti-Mouse (Cell Signaling Technology, #7076, 1:1000). The protein band intensities were quantified using the Bio-Rad Molecular Imager ChemiDoc TM XRS + system.

### RNA extraction, quantitative RT-PCR and RNA sequencing

Total RNA was extracted with an RNA extraction kit (TaKaRa, China) and used to synthesize cDNA using PrimeScript™ RT Master Mix (TaKaRa) according to the manufacturer’s instructions. QRT-PCR was performed with SYBR® Premix Ex Taq™ kit (TaKaRa) using a CFX96 QPCR Detection System (Bio-Rad) in a total reaction volume of 10 μl. 2 − ΔΔCt methods were used to calculate to detect the expression. The primer sequences are listed in Supplemental Table 1. RNA sequencing was performed by the Gene Denovo as previously described (Guangzhou, China) [20].

### Cell counting kit-8 (CCK8) assay and colony formation assay

For cell proliferation, the CCK8 assay was performed. OS cells were transfected with the indicated plasmids, seeded into each well of a 96-well microplate at 1 × 10^3^ cells per well, and incubated for 0–4 days. At different times, the cells were incubated with 10 μl CCK8 reagent (Invitrogen) for 2 h at 37 °C. An optical density of 450 nm relative to a blank well was measured. For colony formation, 500 cells were resuspended and inoculated into a 6-well plate with culture medium. After 14 days at 37 °C, cell colonies were stained with 0.1% crystal violet and imaged under a microscope.

### Cell migration and adhesion assay

For cell migration, transfected OS cells (0.2 ml, 3.5 × 10^5^/ml) were incubated in serum-free medium overnight, then suspended and added into the upper chamber of a transwell (BD, NJ, USA), while the bottom chamber was filled with 700 μl of 10% FBS medium. After 24 h, the cells that migrated through the transwell were fixed and stained with 0.1% crystal violet, then counted under high magnification. For the wound healing assay, OS cells were grown to 80% confluence in 6-well plates and then wounded with a sterile pipette tip. Five photographs were taken for each dish at 0, 24, 48 and 72 h, and the average migration distance in each photograph was calculated by Image-Pro Plus 6.0. The cell adhesion assay was performed with the indicated cells as previously described [20].

### Fluorescence in situ hybridization (FISH)

LINC00662 probes were designed and synthesized by Boster (Wuhan, China). The hybridization and fluorescence detection were conducted with a FISH Detection Kit (Boster) following the manufacturer’s instructions. The image was observed using confocal laser microscopy (Leica, Wetzlar, Germany).

### Multiplex human cytokine ELISA and ELISA

CCL22 levels in the culture medium of THP-1-derived macrophages were measured by ELISA using a ELISA kit (R&D, Minneapolis, USA) according to the instructions of the manufacturer. The multiplex human cytokine ELISA was performed by Asbio (Guangzhou, China).

### Luciferase reporter assay

The wild-type (WT) or STAT3 binding site-mutated (Mut) LINC00662 promoters were cloned into the pGL3 luciferase reporter plasmid (Promega, Madison, WI), then co-transfected with STAT3 or NC, in OS cells, using Lipofectamine 3000. Forty-eight hours later, the luciferase activities were measured by the Dual-Luciferase Reporter Assay System (Promega) according to the manufacturer’s instructions.

### Flow cytometry

Transfected OS cells were inoculated into 6-well plates and resuspended in 1 × binding buffer after 48 h. For the apoptosis assay, 2 μl Annexin V/PI was added to 100 μl of cell suspension and the mixture was incubated for 5 min in the dark. For expression of CD163 in THP-1 cells, FITC-CD163, or IgG (BD Biosciences, San Jose, CA, USA) antibody was added into 100 μl of cell suspension and the mixture was incubated for 15 min in the dark. Then, 400 μl PBS was added and the fluorescence intensity of the cells was analyzed immediately using a BD Accuri TM C6 (BD, NJ, USA) flow cytometer.

### Exosome extraction, identification and uptake

Exosome isolation from culture medium was performed by using ExoQuick-TC for Tissue culture media and Urine kit (SBI, USA) as previously described [20]. The identification of exosomes was performed, by GeneChem (Shanghai, China), using nanoparticle tracking analysis and transmission electron microscopy. Isolated exosomes were stained using PKH26 Red Fluorescent Cell Linker Kits (Sigma, MO, USA) according to the manufacturer’s instructions. PKH26-labeled exosomes or exosomal LINC00662 (identified by GFP fluorescence) were then resuspended and incubated with PMA treated THP-1 cells at 37 °C for 4 h. A laser confocal microscope (LSM710, Zeiss, Germany) was used to observe the fluorescence signal.

### Animal studies

OS cell (5 × 10^6^/50 μl) suspensions were subcutaneously injected into the flank of 5-week-old male athymic BALB/c nude mice. Tumor size was measured every week for a total period of 4 weeks. Tumor volume was determined using the formula: volume = (length × width^2^)/2. To evaluate metastasis, stably-transfected and control OS cells were injected into nude mice through the tail vein as previously described [20].

### Statistical analysis

All data are expressed as mean ± SD and analyzed using SPSS software version 20.0 (SPSS Inc., Chicago, IL). Differences between different treatment groups were assessed by the Student’s t test or one-way ANOVA. Differences were statistically significant when p < 0.05.

## Results

### PITX1 inhibits OS cell proliferation and invasion in vivo and in vitro

**Fig. 1:**
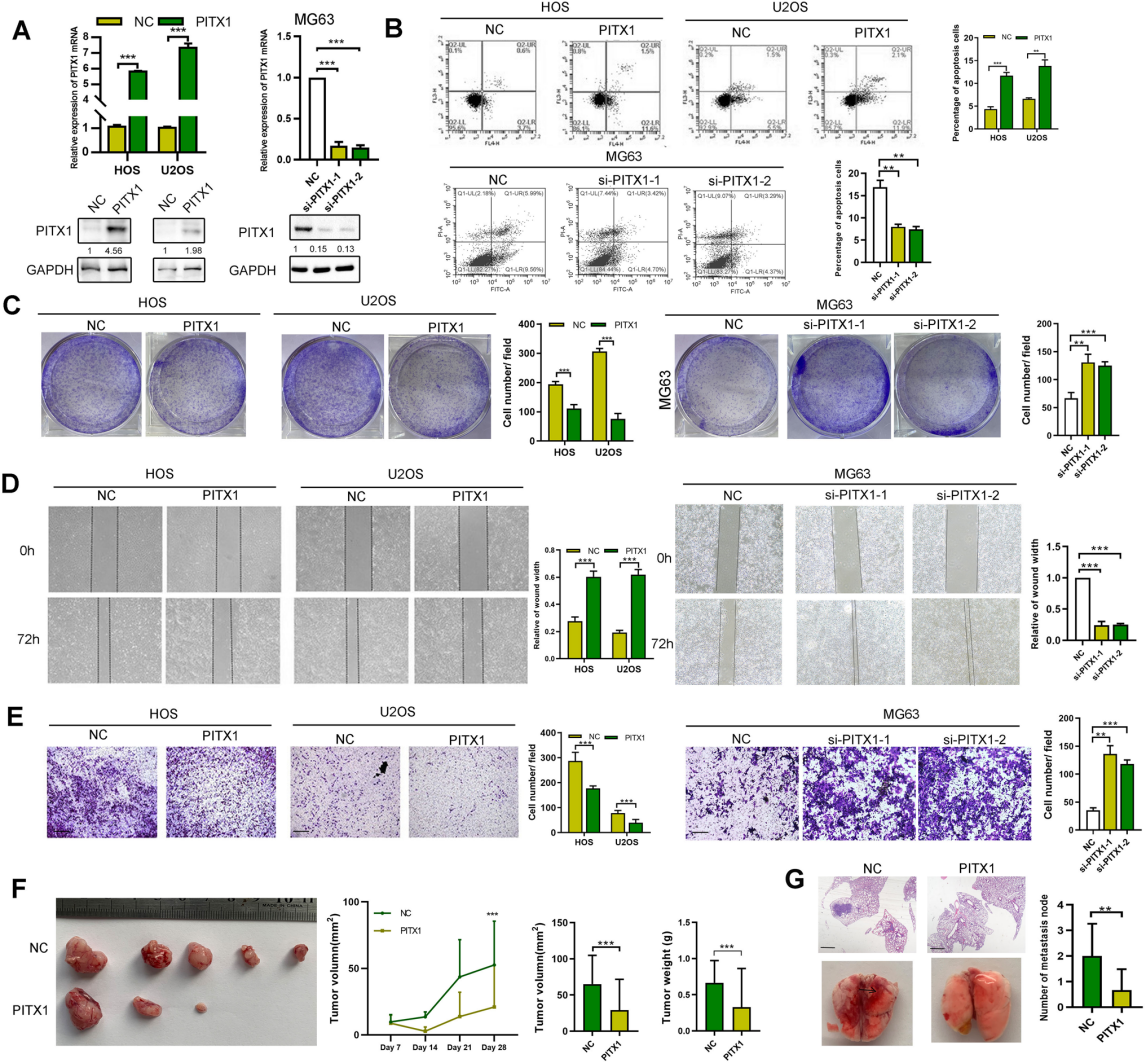
Function of PITX1 in OS in vitro and in vivo. **A** PITX1 expression was detected by qRT-PCR and western blotting following transient transfection of the PITX1 plasmid or two siRNAs in HOS, U2OS and MG63 cells. **B** Apoptosis of PITX1-overexpressing or -knockdown OS cells was measured by Annexin V-PI staining and flow cytometry. Percentage of apoptotic cells (Q2 + Q4) was determined after 48 h following PITX1 plasmid or siRNA transfection. **C** Colony formation of NC and PITX1-overexpressing or -knockdown OS cells was detected. **D** Wound healing assay was performed to measure the migration of NC and PITX1-overexpressing or -knockdown OS cells. **E** Transwell assays were performed to detect the migration of NC and PITX1-overexpressing or -knockdown OS cells. Scale bar, 100 μm. **F** Images of tumors obtained from NC or stable PITX1-overexpressing tumors of BALB/c nude mice after 28 days (n = 5 each group). Tumor volumes were measured every 7 days and the tumor weights were determined at the 28th day after inoculation. **G** The H&E staining images obtained from lung metastatic nodules from NC or PITX1-transfected tumor cells in BALB/c nude mice after 35 days (n = 5 each group). Scale bar, 400 μm. Data represent the mean ± SD of 3 separate determinations. *p < 0.05, **p < 0.01, ***p < 0.001 by Student’s t test

PITX1 is a transcriptional factor located in the nucleus of OS cells (Fig. S1A). We previously showed the expression of PITX1 is down regulated in OS tissues compared with normal tissues [8]. First, we confirmed this result by using western blot and qRT-PCR (Fig. S1B, C). Next, we detected PITX1 expression in OS cells and found that MG63 cells had the highest expression among the four OS cell lines (Fig. S2A). We then transiently transfected U2OS and HOS cells with the PITX1 plasmid, and MG63 cells with PITX1 siRNAs. After 48 h, the transfection efficiency was verified by qPCR and western blot (Fig. 1A). PITX1 transfection induced apoptosis and inhibited proliferation in both U2OS and HOS cells (Figs. 1B, S2B, C). In addition, MG63 cells transfected with PITX1 siRNAs displayed increased proliferation and decreased apoptosis in comparison with control (Fig. 1B, C). PITX1 overexpression repressed colony formation, indicating that PITX1 suppresses the tumorigenic potential of OS cells (Fig. 1C). The migration of OS cells was determined by transwell and wound healing assays. As shown in Fig. 1D, E, PITX1 transfection inhibited whereas knockdown PITX1 enhanced OS cell migration.

To confirm our in vitro findings, we explored the biological role of PITX1 in vivo. U2OS cells stably overexpressing PITX1 were constructed and inoculated subcutaneously into nude mice. Then, the size of the tumors was observed weekly. As shown in Fig. 1F**,** PITX1 dramatically inhibited tumor growth, based on reduced tumor size and weight compared with the control group. To investigate the metastatic potential of PITX1 in vivo, cells were injected into the tail veins of nude mice, followed by harvesting of lung tissues after five weeks. As shown in Fig. 1G, PITX1 effectively decreased the number and size of lung metastatic lesions, as shown by H&E staining. All results demonstrated a tumor suppressor role for PITX1 in OS.

### PITX1 mediates both OS cell adhesion to the extracellular matrix (ECM), and EMT

**Fig. 2:**
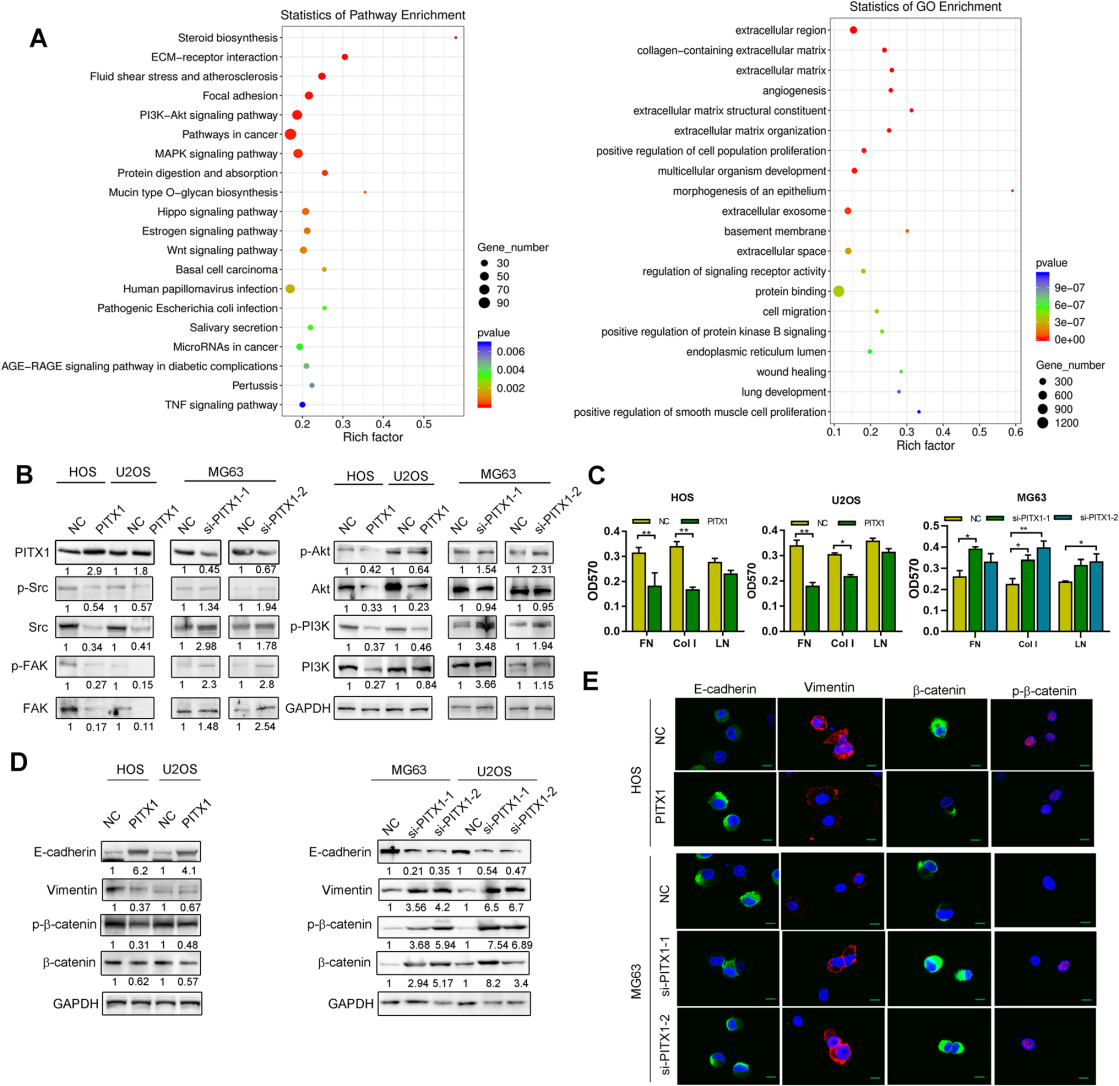
PITX1 regulates EMT and cell adhesion of OS cells. **A** RNA-Seq was performed to identify the differentially-expressed genes (DEGs) between NC and PITX1-overexpressing U2OS cells. KEGG analysis was used to show the relationship of overlapping DEGs and pathways. GO analysis showed that the DEGs were enriched in extracellular matrix. **B** Expression of FAK/Src and PI3K/Akt signaling pathway mediators was determined by western blotting in PITX1-overexpressing or -knockdown OS cells. **C** Cell adhesion was measured in NC and PITX1-overexpressing OS cells on plates coated with fibronectin (FN), laminin (LN), and collagen I (Col I). **D** Expression of EMT biomarkers in NC and PITX1-overexpressing or -knockdown OS cells was detected by western blotting. **E** Expression of EMT biomarkers in NC and PITX1-overexpressing or -knockdown was detected by immunofluorescence (Scale bars: 20 μm). Data represent the mean ± SD of three separate determinations. *p < 0.05, **p < 0.01, ***p < 0.001 by Student’s t test

We sought the mechanisms of the anti-tumor effects induced by PITX1 by screening its targets genes using RNA sequencing. There were 1761 upregulated and 1057 downregulated differentially-expressed genes (DEGs) between control and PITX1-overexpressing U2OS cells (Fig. 2A). DEG analysis showed that extracellular matrix and angiogenesis were ranked as the top enriched Gene Ontology (GO) terms in biology process. Furthermore, the Kyoto Encyclopedia of Genes and Genomes (KEGG) pathway analysis categorized ECM, focal adhesion and PI3K/Akt in the top 20 pathways. Western blotting confirmed that the expression and phosphorylation of focal adhesion kinase (FAK) and steroid receptor coactivator (Src) kinases, as well as phosphoinositide 3-kinase (PI3K) and protein kinase B (Akt) proteins, were elevated by PITX1 knockdown. Conversely, they were significantly repressed in PITX1-overexpressing cells (Fig. 2B).

To test whether PITX1 destabilizes cancer cell adhesion to the ECM, we performed cell adhesion assays by seeding control or PITX1-overexpressing OS cells in plates coated with defined components of the ECM, i.e., fibronectin, laminin, and collagen I. The results showed that PITX1 inhibited cell adhesion of OS cells (Fig. 2C). Then, we investigated whether PITX1 regulated the expression of EMT biomarkers in OS and found that PITX1 induced a significant increase in E-cadherin expression and an reduction in p-β-catenin and vimentin expression in OS cells, whereas PITX1 knockdown had produced the opposite results (Fig. 2D). These results were confirmed by IF staining (Fig. 2E). These data suggested that PITX1 reduces OS cell adhesion and EMT.

### PITX1 mediates OS malignancy via suppressing LINC00662

**Fig. 3:**
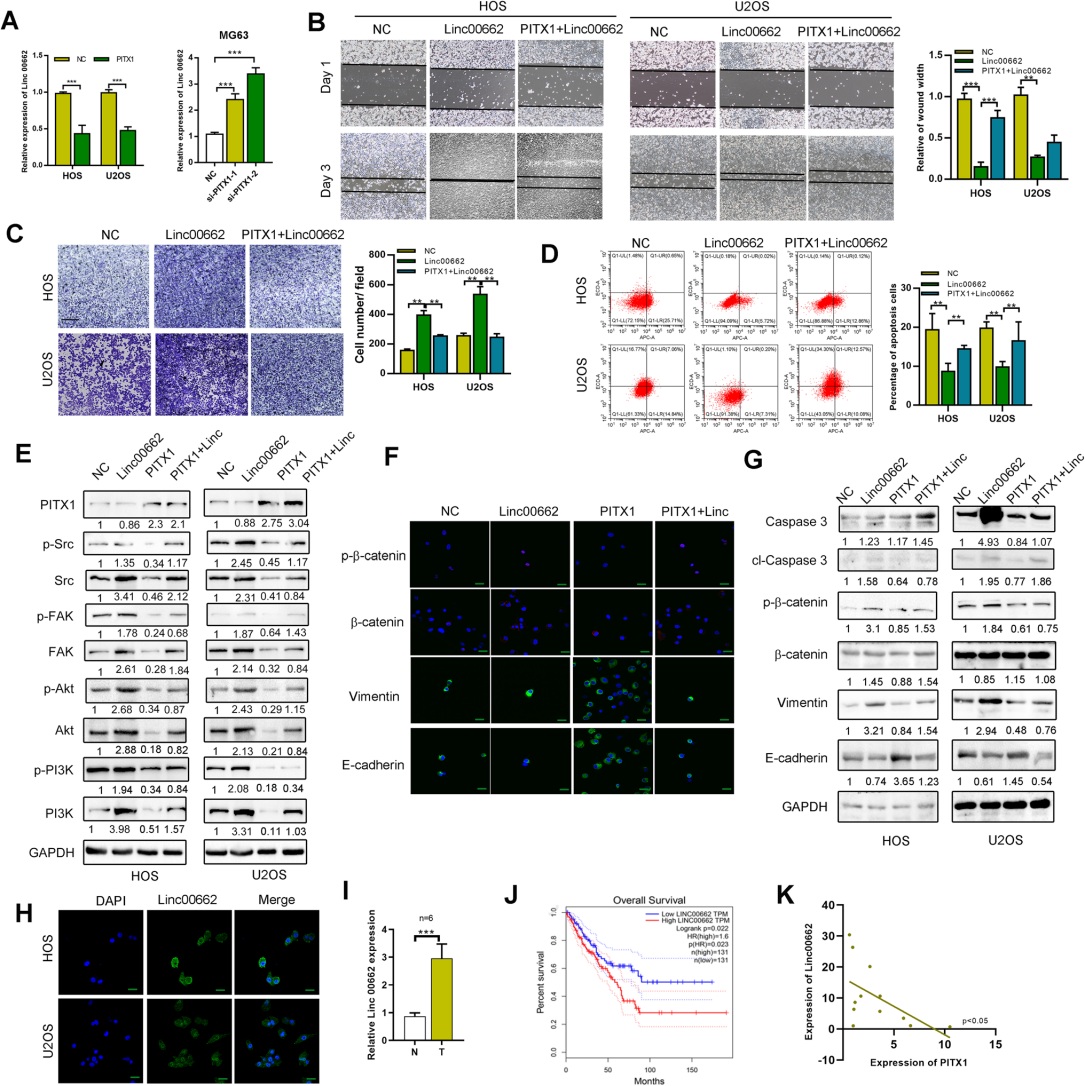
PITX1 regulates OS function via LINC00662. **A** Expression of LINC00662 was detected by qRT-PCR in NC and PITX1-overexpressing or -knockdown OS cells.** B** A wound healing assay was performed to measure the cell migration of NC, LINC00662-, and PITX1-, LINC00662-overexpressing OS cells. **C** Transwell assay was performed to measure the cell migration of NC, LINC00662-, and PITX1-, LINC00662-overexpressing OS cells. Scale bar, 100 μm. **D** Percentage of apoptotic cells was determined by Annexin V-PI staining and flow cytometry in NC, LINC00662-, and PITX1-, LINC00662-overexpressing OS cells. Percentage of apoptotic cells (Q2 + Q4) was determined 48 h after transfection. **E** Expression of FAK/Src and PI3K/Akt signaling pathway mediators were detected by western blotting in NC, LINC00662- and PITX1-, LINC00662-overexpressing cells. **F** Expression of EMT biomarkers was detected in HOS cells by immunofluorescence (Scale bars: 20 μm). **G** Expression of apoptosis and EMT biomarkers was detected by western blotting in HOS and U2OS cells. **H** Location of LINC00662 in OS cells was detected by FISH (Scale bars: 20 μm). **I** Expression of LINC00662 was detected in OS tissues (n = 23) and normal specimens (n = 7). **J** Survival curve of OS patients with different LINC00662 expression levels was determined using TCGA. **K** The correlation between PITX1 and LINC00662 mRNA expression in OS tissues (n = 12) is shown. Data represent the mean ± SD of three separate determinations. *p < 0.05, **p < 0.01, ***p < 0.001 by Student’s t test

Among the DEGs between control and PITX1-overexpressing U2OS cells, LINC00662 has been shown to exert an oncogenic effect in OS [21, 22]. Thus, we further examined the ability of PITX1 to regulate the expression of LINC00662. Our qRT-PCR results showed that LINC00662 expression was significantly downregulated in PITX1-overexpressing OS cells and was upregulated in cells following PITX1 knockdown, the transfection efficiency of PITX1 was confirmed by qRT-PCR (Figs. 3A, S3A, B). As shown in Fig. S3C–F, high expression of LINC00662 endowed HOS and U2OS cells with strong migratory and colony forming ability. Then, we examined whether LINC00662 could reverse PITX1-induced tumor suppression. In wound healing and transwell assays using U2OS and HOS cells, LINC00662-enhanced cell migration was reversed by PITX1 overexpression (Fig. 3B, C). Moreover, LINC00662-inhibited cell apoptosis was enhanced by PITX1 transfection, as judged by flow cytometry (Fig. 3D). Importantly, immunofluorescence and western blotting showed the expression of FAK/Src and PI3K/Akt pathway regulators and EMT biomarkers were also mediated by LINC00662 in PITX1-overexpressing cells (Fig. 3E–G). Then, we examined the correlation between PITX1 and LINC00662 expression. LINC00662 was located both in the nucleus and cytoplasm, as detected by FISH (Fig. 3H). LINC00662 expression was markedly higher in tumor tissues (n = 23) compared with the normal tissues (n = 7) (Fig. 3I). Patients with high LINC00662 expression had shorter survival compared with patients with low LINC00662 expression, based on the patient population in TCGA (Fig. 3J). By qRT-PCR, the expressions of PITX1 and LINC00662 in 12 OS tissues were negatively correlated (Fig. 3K). These results indicate that PITX1 inhibits OS malignancy by downregulating LINC00662.

### PITX1 regulates LINC00662 via suppressing transcription factor STAT3 expression

**Fig. 4:**
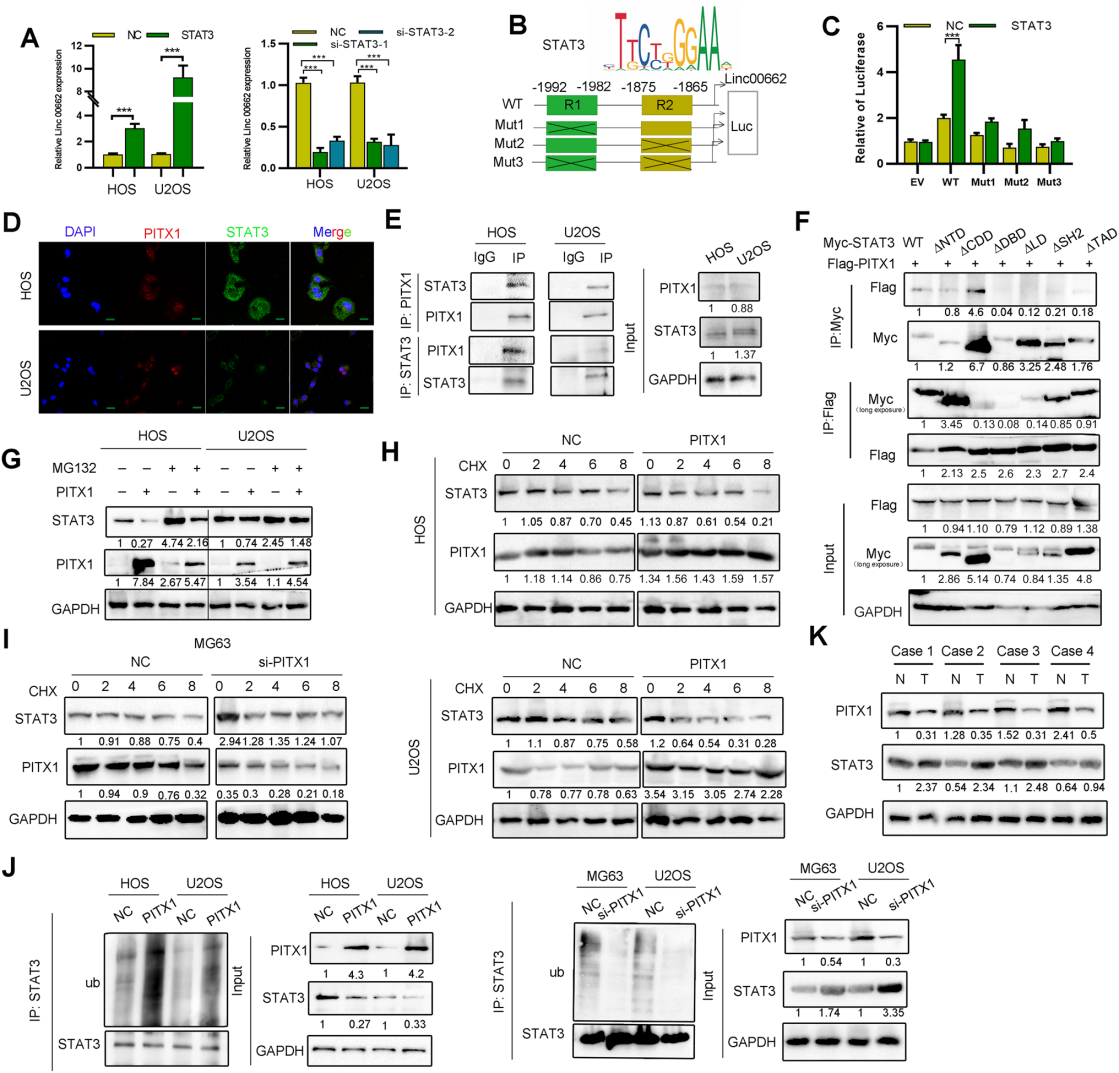
PITX1 promotes STAT3 proteasome degradation to inhibit LINC00662 expression. **A** Expression of LINC00662 was detected after STAT3 overexpression or knockdown in OS cells. **B** Online JASPAR software was used to predict the possible binding sites (R1 and R2) of STAT3 in the LINC00662 promoter. Schematic diagram of luciferase reporter vectors containing wild-type (WT) or mutant (Mut1: R2 mutant, Mut2: R1 mutant, Mut3: double R1/R2 mutant) LINC00662 promoters. **C** Dual luciferase reporter assay was conducted in OS cells transfected with wild-type or mutant reporters and control vector. **D** Co-localization of PITX1 and STAT3 was detected by immunofluorescence (Scale bars: 20 μm). **E** Binding of STAT3 and PITX1 was detected by co-IP in HOS and U2OS cells. **F** Binding of different STAT3 domains and PITX1 were detected by co-IP using HEK 293 T cells. **G** Expression of STAT3 was detected in PITX1-overexpressing cells following MG132 treatment (10 μm). **H** Expression of STAT3 was detected by western blotting in control and PITX1-overexpressing OS cells treated with CHX (10 ng/ml) for different times. **I** Expression of STAT3 was detected by western blotting in control and PITX1-knockdown OS cells treated with CHX (10 ng/ml) for different times. **J** Immunoprecipitation showing the ubiquitination of STAT3 by ectopic expression of PITX1. STAT3 ubiquitination was detected by immunoprecipitation with anti-STAT3, and then immunoblotted with the indicated antibodies. **K** Expression of STAT3 and PITX1 in OS and normal tissues was detected by western blotting (n = 4 pairs). Data represent the mean ± SD of three separate determinations. *p < 0.05, **p < 0.01, ***p < 0.001 by Student’s t test

Previous research indicates that transcription factor STAT3 mediates LINC00662 expression by binding to its promoter [23]. Thus, we speculated that PITX1 could mediate LINC00662 expression via STAT3. QRT-PCR showed that LINC00662 expression was enhanced upon STAT3 upregulation (Fig. 4A). The GEPIA website also identified a positive correlation between STAT3 and LINC00662 in sarcoma (Fig. S4A). There are two putative binding sites for STAT3 in the LINC00662 promoter as predicted by JASPAR (Fig. 4B). ChIP and luciferase reporter assays revealed that STAT3 occupied sites at −1992 ~  −1982 and −1875 ~ −1865 in the LINC00662 promoter (Figs. 4C, S4B). These results suggest that STAT3 contributes to the transcriptional activation of LINC00662.

We previously showed that STAT3 stability is regulated through ubiquitin-dependent proteasomal degradation [19]. First, immunocytochemical staining showed that PITX1 and STAT3 were co-localized in the nucleus of OS cells (Fig. 4D). Subsequently, co-IP further confirmed that PITX1 and STAT3 bound to each other directly in OS cells via the DNA binding domain of STAT3 (Fig. 4E, F). Of note, overexpression of PITX1 decreased the expression levels of STAT3. However, this decrease was blocked by treatment with the proteasome inhibitor MG132, suggesting protein stability may contribute to the PITX1-mediated STAT3 downregulation (Fig. 4G). Furthermore, U2OS cells treated with the protein synthesis inhibitor cycloheximide (CHX) showed that PITX1 overexpression decreased STAT3 expression by shortening its half-life (Fig. 4H). Conversely, PITX1 knockdown prolonged the half-life of STAT3 to stabilize its expression (Fig. 4I). As expected, overexpression of PITX1 elevated the polyubiquitination of STAT3 (Fig. 4J), and the expressions of PITX1 and STAT3 were negatively correlated with each other in OS patient samples (Fig. 4K). Overall, these results indicate that PITX1 mediates the expression of STAT3 in a proteasome-dependent manner.

### PITX1 mediates macrophage M2 polarization via OS cell-derived exosomal LINC00662

**Fig. 5:**
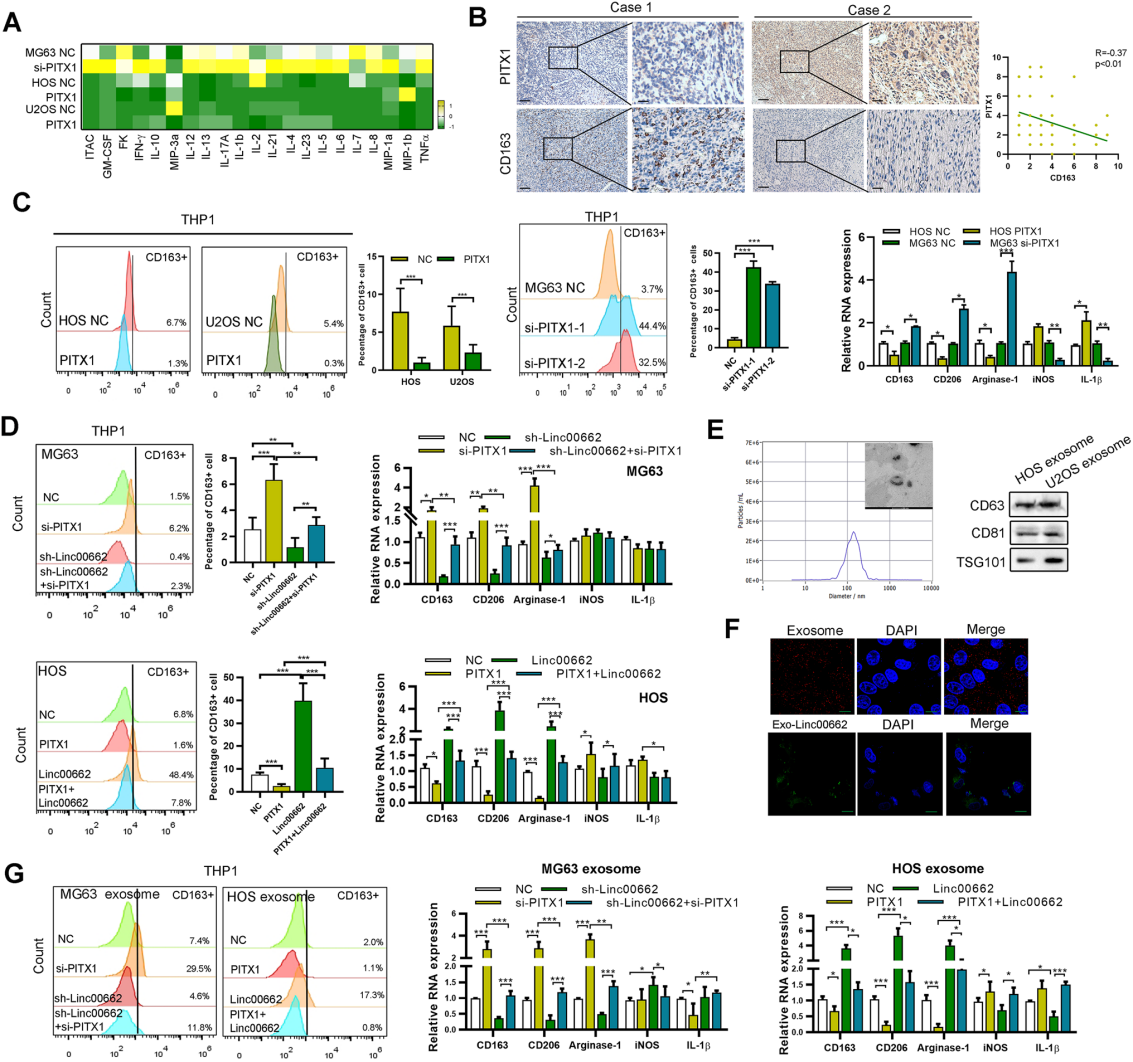
PITX1/LINC00662 regulates M2 macrophage polarization. **A** Multiple cytokine ELISA was performed to detect the secreted cytokines in control and PITX1-transfected or -knockdown OS cells. The heatmap shows the difference between each group. **B** Expression of CD163 and PITX1 was detected by immunohistochemistry in OS tissues (Scale bars: 100 μm, 50 μm). The correlation between CD163 and PITX1 is shown. **C** PMA-treated THP-1 cells were co-cultured with NC, PITX1-overexpressing or PITX1-knockdown OS cells, the expression of M2 macrophage biomarkers (CD163, CD206 and arginase-1) and M1 macrophage (iNOS and IL-1β) were determined qRT-PCR. The expression of CD163 was detected by flow cytometry. **D** PMA-treated THP-1 cells were co-cultured with NC, PITX1 knockdown, LINC00662 knockdown or both knockdown OS cells. PMA-treated THP-1 cells were co-cultured with NC, PITX1-overexpressing, LINC00662 overexpressing or both overexpressing OS cells. The expression of M1 and M2 macrophage biomarkers were determined by qRT-PCR, and the expression of CD163 were detected by flow cytometry. **E** Exosomes derived from control and LINC00662 HOS cells were identified by using Nano Sight nanoparticle tracking, electron microscopy and western blot analysis (Scale bars: 200 nm). **F** Representative immunofluorescence image showing internalization of PKH26-labeled, HOS-derived exosomes (red) and exosomal GFP-labeled LINC00662 (green) by PMA-treated THP-1 cells (Scale bars: 20 μm). **G** PMA-treated THP-1 cells were co-cultured with exosomes derived from NC or PITX1-overexpressing or -knockdown OS cells, or with LINC00662-overexpressing or -knockdown OS cells. Expression of M1 and M2 macrophage biomarkers were determined qRT-PCR, and the expression of CD163 were detected by flow cytometry. Data represent the mean ± SD of three separate determinations. *p < 0.05, **p < 0.01, ***p < 0.001 by Student’s t test. (Color figure online)

Previous reports showed that LINC00662 could promote M2 macrophage polarization in hepatocellular carcinoma [12], thus we further explored whether PITX1/LINC00662 could mediate macrophage polarization to affect OS malignancy. Using a multiplex human cytokine ELISA, we found that knockdown of PITX1 elevated the expression of macrophage inflammatory proteins (MIPs), while overexpressing PITX1 decreased MIP expression in HOS and U2OS cell lines (Fig. 5A). Furthermore, we found that CD163 + macrophages were more abundant in the stroma of the OS tissues compared to the stroma of normal tissues (Fig. S5A**)**. The expression of PITX1 detected by immunohistochemistry was negatively correlated with CD163, a biomarker of macrophage M2 polarization (Fig. 5B). To further explore whether PITX1-overexpressing OS cells could induce M2 polarization of macrophages via LINC00662, we used PMA to differentiate THP-1 cells into M0 macrophages and co-cultured the differentiated THP-1 cells with LINC00662-transfected OS cells. We found that the LINC00662-transfected cells could induce M2 polarization, as defined by elevated expression of the M2 macrophage biomarkers (CD206, arginase-1 and CD163) detected by qRT-PCR and the expression of the typical M2 marker CD163 detected by flow cytometry (Fig. S5B). Then we co-cultured the PITX1-knockdown OS cells with PMA-induced THP-1 cells and found that co-culture with PITX1-knockdown cells could elevate the expression of M2 macrophage biomarkers while reducing expression of M1 macrophage markers (IL-1β and iNOS) detected by qRT-PCR. Expression of the typical M2 marker CD163 was increased in the PITX1-knockdown co-culture group compared with the control vector transfection group, detected by flow cytometry. As expected, PITX1-transfected cells gave the opposite results (Figs. 5C, S5C). However, the ability of PITX1-overexpressing cells to inhibit macrophage M2 polarization was visibly reversed by LINC00662-transfected OS cells (Fig. 5D).

Exosome-mediated lncRNA delivery is widely believed to contribute to M2 polarization in many cancers [12, 24]. Thus, we speculated that OS cell-derived exosomes could transfer LINC00662 to THP1 cells. As shown in Fig. S5D**,** PMA-differentiated THP-1 cells incubated with exosomes derived from LINC00662-overexpressing OS cells exhibited high LINC00662 expression compared to that incubated with exosomes from control OS cells, and could be prevented by the exosome inhibitor GW4869. Exosomes derived from OS cell lines were isolated and purified from the culture medium. Electron microscopy nanoparticle tracking and western blot analysis confirmed that the isolated particles were exosomes (Fig. 5E). Then, we labeled the OS cell-derived exosomes with PKH26 and added them to PMA-differentiated THP1 cells. The exosomes were internalized by the unstained THP-1 cells (Fig. 5F). Exosomes were extracted from OS cells transfected with GFP-LINC00662 and added to PMA-differentiated THP1 cells. Similar to the PKH26-labeled exosomes, the exosomal GFP-LINC00662 was internalized by PMA-induced THP-1 cells (Fig. 5F). The expression of LINC00662 was higher in PMA-induced THP1 cells co-cultured with LINC00662-containing exosomes than in exosomes derived from control vector transfection cells (Fig. S5E). Consistent with the above results, the expression levels of M2 macrophage biomarkers (CD206, arginase-1 and CD163) detected by qRT-PCR, as well as the expression of the typical M2 marker CD163 detected by flow cytometry, were significantly increased in PMA-induced THP1 cells treated with exosomes derived from OS cells overexpressing LINC00662 or underexpressing PITX1, compared with THP1 cells treated with exosomes derived from control vector transfection OS cells, while reversed by PITX1 transfection or LINC00662 knockdown (Figs. 5G, S5F).These results indicate that PITX1-knockdown OS cells induce macrophage M2 polarization via OS cell-derived exosomal LINC00662.

### LINC00662-induced M2 macrophages regulate OS cell EMT and migration via secretion of CCL22

**Fig. 6:**
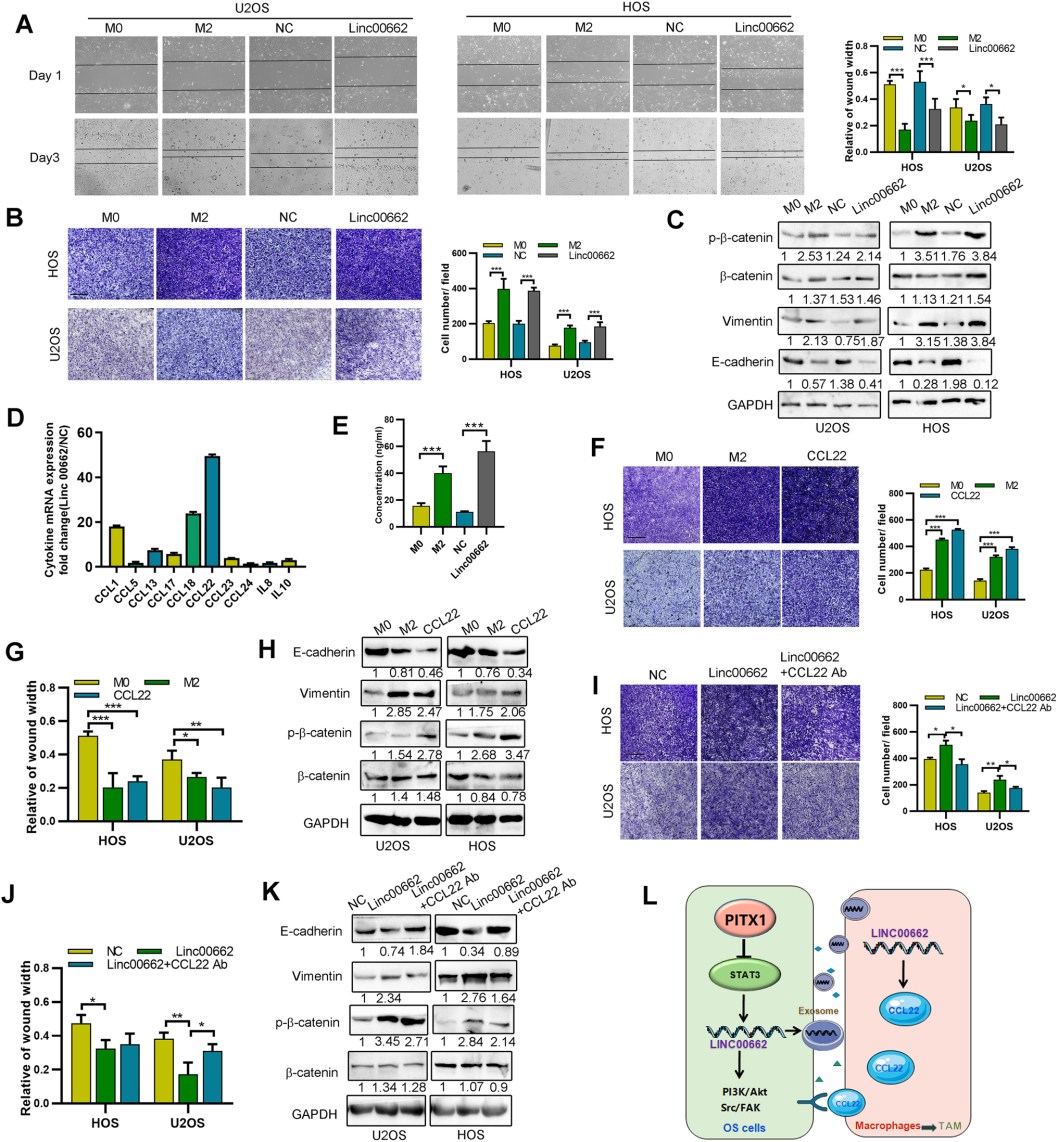
CCL22 is responsible for the pro-EMT effect of LINC00662-induced macrophages.** A** A wound healing assay was performed using LINC00662-transfected or NC-transfected THP-1-treated OS cells. OS cells were co-cultured with M0 or M2 macrophages. **B** Transwell assay was performed using the OS cells co-cultured with the indicated macrophages. Scale bar, 100 μm. **C** Western blot analyses of EMT biomarker expression were performed using OS cells co-cultured with the indicated macrophages. **D** Relative expression of cytokine mRNA expression levels in LINC00662-transfected macrophages versus control macrophages was detected by qRT-PCR. **E** Secreted CCL22 levels in M0, M2, control or LINC00662-transfected macrophages conditioned media determined by ELISA. **F** Transwell assay was used to measure the migration of CCL22-treated OS cells, and OS cells co-cultured with M0 or M2 macrophages. Scale bar, 100 μm. **G** A wound healing assay was used to measure the migration of CCL22-treated OS cells, and OS cells co-cultured with M0 or M2 macrophages. **H** Western blot analyses of EMT biomarker expression in the indicated cells. **I** Transwell assays were used to measure the migration of OS cells co-cultured with control or LINC00662-transfected macrophages with or without anti-CCL22 antibody. Scale bar, 100 μm. **J** A wound healing assay was used to determine the migration of OS cells co-cultured with control or LINC00662-transfected macrophages with or without anti-CCL22 antibody. **K** Western blot analyses of EMT biomarker expression in OS cells co-cultured with control or LINC00662-transfected macrophages with or without anti-CCL22 antibody. **L** Schematic diagram of the project

M2 macrophages have been reported to enhance cell migration and EMT in various cancers via secreting anti-inflammatory cytokines [25, 26]. First, we found that the migrating and adhering ability of OS cells was significantly increased after co-culture with M2 macrophages or LINC00662-overexpressing THP1 cells, compared with M0 macrophages or control vector transfection cells (Fig. 6A, B). These M2 macrophages also promoted the expression of mesenchymal biomarkers and down regulated the expression of epithelial biomarkers detected by western blotting (Fig. 6C). Then, we detected the expression of several typical secreted inflammatory cytokines in OS cell-derived LINC00662 exosome-treated M2 macrophages and found that CCL22 was highly expressed (Fig. 6D). These results were subsequently supported by ELISA (Fig. 6E). Then we found CCL22 enhanced both the migration and expression of mesenchymal biomarkers in OS cells, an effect similar to that induced by M2 macrophages (Fig. 6F–H). To determine whether LINC00662-induced M2 macrophage CCL22 was responsible for the enhanced OS cell migration and EMT, we employed an anti-CCL22 antibody to neutralize the function of CCL22. We found that the elevation of OS cell migration, resulting from co-culture with LINC00662-induced M2 macrophages, was inhibited by anti-CCL22 antibody (Fig. 6I, J). Moreover, we found that CCL22 antibody reversed the expression of EMT biomarkers induced by M2 macrophages (Fig. 6K). Together, these results indicate that LINC00662-induced M2 macrophages promote the migration and EMT of OS cells via CCL22.

## Discussion

OS is the most common form of primary bone malignancy with poor prognosis due to lung metastasis and chemotherapeutic resistance [1]. First-line therapy, such as doxorubicin, cisplatin and high-dose methotrexate, alongside limb-sparing surgery, is used for most OS patients [27]. However, more effort is needed to increase OS patient survival by identifying candidate therapies via experimental studies and clinical trials of novel treatments. In this paper, we demonstrate that PITX1 inhibits OS cell proliferation and migration via suppression of LINC00662 expression. PITX1 increases proteasomal degradation of STAT3, a transcription factor for *LINC00662*. Importantly, we show that LINC00662-enriched exosomes promote M2 macrophage polarization by potentially mediating the crosstalk between OS cells and TAMs within the metastatic microenvironment (Fig. 6L).

We previously showed that PITX1 expression is downregulated in OS compared with normal tissues. High expression of PITX1 is associated with better patient survival, consistent with findings in other types of cancer [3, 4, 8, 28]. For example, PITX1 expression is decreased in breast cancer, lung cancer and Barrett’s adenocarcinoma relative to their normal tissues [3, 5, 29]. The biological function of PITX1 has been demonstrated in several types of cancer. For example, in melanoma, unregulated PITX1 results in a reduction in cell proliferation and an increase in apoptosis [30]. However, the function of PITX1 in OS has not been demonstrated. Thus, we further characterized the function of PITX1 in OS and found that high expression of PITX1 prominently inhibits cell proliferation, colony formation, cell migration and invasion, and increases apoptosis. These results indicate that PITX1 regulates the biological function of OS cells.

RNA-Seq analysis showed LINC00662, an oncogenic lncRNA in tumors, to be a downstream target of PITX1. The biological functions of LINC00662 in several human cancers have been demonstrated recently. LINC00662 is highly expressed and promotes malignant progression in several types of cancer, including gastric cancer, lung cancer, and oral squamous cell carcinoma [13, 31]. Previous studies demonstrated the possibility of LINC00662 as a therapeutic target for chordoma, lung cancer and breast cancer [13]. Mechanistically, LINC00662 exerts oncogenic functions by targeting the miR-16-5p/ITPR1 and miR-103a-3p/SIK2 axis in OS [21, 22]. In the present study, we found that LINC00662 promotes OS cell proliferation and migration in vitro. Previous studies combined with our findings together provide strong evidence to support LINC00662 as an important tumor promoter and promising therapeutic target of OS.

Emerging evidence states that transcription factors, such as Oct4, bind to promoters and induce the expression of lncRNAs [32]. With the assistance of bioinformatic prediction tools, as well as ChIP and luciferase reporter assays, STAT3 was identified as a transcription factor responsible for LINC00662 up-regulation in OS cells by binding to the *LINC00662* promoter region, consistent with a previous report in glioma [23]. STAT3, a highly conserved transcription factor, has also been reported to be overexpressed in various types of cancer and promotes carcinogenesis through modulating cell proliferation, apoptosis, EMT, metastasis and drug resistance [33]. In accordance with previous reports concerning STAT3 in OS [34], we found that STAT3 expression is up-regulated in OS tumors compared with normal tissue. More importantly, STAT3 expression is negatively correlated with PITX1 expression in OS tissues. Through a series of bioinformatic predictions and experimental validation, we identified STAT3 as a binding partner of PITX1, and mechanistically, PITX1 serves as a mediator for STAT3 proteasome degradation. Since STAT3 has been validated as an anticancer target, inhibition of STAT3 activity has been considered as a promising approach for cancer therapy [35]. However, currently there is no STAT3 targeted drug approved for clinical application.

Recent research has shown that the TME also plays critical roles in tumor initiation and progression. Crosstalk between the TME and cancer cells could mediate cancer cell proliferation, invasion, apoptosis, and angiogenesis, as well as immune evasion [36]. Evidence indicates that TAMs, especially the M2-polarized subtype, play a key role in regulating tumor growth, migration, and angiogenesis [37]. Our data suggests that PITX1 mediates MIP expression in a paracrine manner. Previous studies state that LINC00662 promotes M2 polarization in hepatocellular carcinoma [12]. Thus, we hypothesized that PITX1/LINC00662 could promote M2 macrophage polarization. The regulatory roles of PITX1 on LINC00662 and M2 macrophage polarization were verified in OS and THP1 cell models in vitro. The correlations between PITX1, LINC00662 and M2 macrophage polarization were further found in clinical OS tissues, which support the modulation of M2 macrophage polarization by PITX1/LINC00662. The possible mechanisms by which M2 polarization promotes tumor progression and regulates TME involve secretion of growth factors and cytokines, such as IL6 and CCL18 [36]. The chemokine CCL22, produced by macrophages, also plays a critical role in immune and inflammatory responses in the biology of cancer cells, and contributes to the development and progression of a number of human cancers [38]. To date, many studies have demonstrated a key role of CCL22 in the malignant progress. For example, CCL22 promotes 5-FU-mediated CRC chemoresistance and EMT through PI3K/AKT signaling, and contributes to poor prognosis in colorectal cancer [39]. In malignant pleural effusion, macrophage-derived CCL22 promotes an immunosuppressive tumor microenvironment via IL-8 [40]. Here, we show that CCL22 secreted by M2 macrophages can promote the migration and EMT of OS cells, and this effect could be reversed by applying the CCL22 antibody.

In conclusion, our data suggest a tumor suppressor role of PITX1 in OS progression. Functionally, PITX1 inhibits OS cell migration, invasion and EMT in vitro and lung metastasis in vivo. Mechanistically, PITX1 decreased LINC00662 expression via promoting STAT3 protein degradation. Exosomal LINC00662-induced M2 macrophages can promote OS cell migration and EMT through CCL22. Our findings also suggest that PITX1 could expand our understanding of OS metastasis and may be a potential therapeutic target for OS.

### Supplementary Information

Below is the link to the electronic supplementary material.

Supplementary file1 (TIF 64397 kb)**—Fig. 1 A** Immunofluorescence and western blotting showing PITX1 was mainly located in the nucleus (Scale bars: 20 μm).** B** Expression of PITX1 in OS and normal tissues was detected by western blotting (n=7 pairs). **C **Expression of PITX1 in OS and normal tissues was detected by qRT-PCR (n=9)

Supplementary file2 (TIF 61306 kb)**—Fig. 2 A **Expression of PITX1 was detected by western blotting in OS cells. **B **Cell proliferation of PITX1-overexpressing or NC cells was performed using a CCK8 assay.** C** Expression of apoptosis biomarkers of PITX1 or NC cells was detected using western blotting

Supplementary file3 (TIF 12084 kb)**—Fig. 3** **A** Differentially-expressed genes of NC and PITX1-transfected cells are shown in a heatmap. **B** Transfection efficiency was confirmed by qRT-PCR showing expression of PITX1 in NC and PITX1-overexpressing or -knockdown OS cell lines.** C **Expression of LINC00662 in NC or LINC00662 stably-expressing HOS and U2OS cells. **D** Transwell assay of NC or LINC00662 stably-expressing HOS and U2OS cells. **E **Colony formation of NC or LINC00662 stably-expressing HOS and U2OS cells. **F **Wound healing assay performed on NC or LINC00662 stably-expressing HOS and U2OS cells

Supplementary file4 (TIF 60292 kb)**—Fig. 4 A **Correlation of STAT3 and LINC00662 expression in TCGA sarcoma cases.** B** ChIP assay was used to determine the binding of STAT3 at the LINC00662 promoter at the predicted sites (R1 and R2)

Supplementary file5 (TIF 8920 kb)**—Fig. 5 A** Expression of CD163 in normal (n=5) and OS tissues (n=45; Scale bars: 100 μm). **B** PMA-treated THP-1 cells were co-cultured with HOS cells transfected with a control or LINC00662 expression vector. Expression of CD163 was determined using flow cytometry and expression typical M2 markers were detected by qPCR. **C** PMA-treated THP-1 cells were co-cultured with NC, PITX1-overexpressing OS cells, the expression of M2 macrophage biomarkers (CD163, CD206 and arginase-1) and M1 macrophage (iNOS and IL-1β) were determined qRT-PCR.** D** PMA-treated THP-1 cells were co-cultured with NC or LINC00662-overexpressing or GW4892-treated OS cells. Expression of CD163 was determined using flow cytometry and expression typical M2 markers were detected by qPCR. **E** Expression of LINC00662 in PMA-treated THP-1 cells co-cultured with LINC00662-overexpressing or control OS cells.** F** PMA-treated THP-1 cells were co-cultured with exosomes derived from NC or PITX1-overexpressing or -knockdown OS cells, or with LINC00662-overexpressing or -knockdown OS cells. Expression of CD163 was determined using flow cytometry

Supplementary file6 (DOCX 16 kb)

## Abbreviations

Akt: Protein kinase B
CCK-8: Cell counting kit-8
CCL22: C–C motif chemokine 22
CHX: Cycloheximide
ChIP: Chromatin immunoprecipitation
DAPI: 4’,6-Diamidino-2-phenylindole
DEG: Differentially expressed gene
DMEM: Dulbecco’s modified eagle’s medium
ECM: Extracellular matrix
EMT: Epithelial-mesenchymal transition
FAK: Focal adhesion kinase
FBS: Fetal bovine serum
GAPDH: Glyceraldehyde-3-phosphate dehydrogenase
GO: Gene ontology
IHC: Immunohistochemistry
IP: Immunoprecipitation
NC: Negative control
MG132: Carbobenzoxy-Leu-Leu-leucinal
OS: Osteosarcoma
PBS: Phosphate-buffered saline
PMA: Phorbol 12-myristate 13-acetate
PI3k: Phosphoinositide 3-kinase
PITX1: Paired-like homeodomain transcription factor 1
qRT-PCR: Quantitative reverse transcription polymerase chain reaction
Src: Steroid receptor coactivator
STAT3: Signal transducer and activator of transcription 3
TAM: Tumor associated macrophage

## References

[1] Whelan JS, Davis LE (2018). Osteosarcoma, chondrosarcoma, and chordoma. J Clin Oncol.

[2] Nemec S, Luxey M, Jain D, Huang Sung A, Pastinen T, Drouin J (2017). Pitx1 directly modulates the core limb development program to implement hindlimb identity. Development.

[3] Otsubo T, Yamada K, Hagiwara T, Oshima K, Iida K, Nishikata K, Toyoda T, Igari T, Nohara K, Yamashita S (2017). DNA hypermethyation and silencing of PITX1 correlated with advanced stage and poor postoperative prognosis of esophageal squamous cell carcinoma. Oncotarget.

[4] Shen X, Gu Y, Yu S, Gong P, Mao Y, Li Y, Zheng Y, Qiao F, Zhao Z, Fan H (2019). Silenced PITX1 promotes chemotherapeutic resistance to 5-fluorocytosine and cisplatin in gastric cancer cells. Exp Ther Med.

[5] Song X, Zhao C, Jiang L, Lin S, Bi J, Wei Q, Yu L, Zhao L, Wei M (2018). High PITX1 expression in lung adenocarcinoma patients is associated with DNA methylation and poor prognosis. Pathol Res Pract.

[6] Wang Q, Zhao S, Gan L, Zhuang Z (2020). Biosci Rep.

[7] Qi DL, Ohhira T, Fujisaki C, Inoue T, Ohta T, Osaki M, Ohshiro E, Seko T, Aoki S, Oshimura M (2011). Identification of PITX1 as a TERT suppressor gene located on human chromosome 5. Mol Cell Biol.

[8] Kong G, Liu Z, Wu K, Zhang Y, Deng Z, Feng W, Chen S, Wang H (2015). Strong expression of paired-like homeodomain transcription factor 1 (PITX1) is associated with a favorable outcome in human osteosarcoma. Tumour Biol.

[9] Yang Z, Li X, Yang Y, He Z, Qu X, Zhang Y (2016). Long noncoding RNAs in the progression, metastasis, and prognosis of osteosarcoma. Cell Death Dis.

[10] Li S, Wang X (2021). The potential roles of exosomal noncoding RNAs in osteosarcoma. J Cell Physiol.

[11] An C, Hu Z, Li Y, Zhao P, Liu R, Zhang Q, Zhu P, Li Y, Wang Y (2022). LINC00662 enhances cell progression and stemness in breast cancer by MiR-144-3p/SOX2 axis. Cancer Cell Int.

[12] Tian X, Wu Y, Yang Y, Wang J, Niu M, Gao S, Qin T, Bao D (2020). Long noncoding RNA LINC00662 promotes M2 macrophage polarization and hepatocellular carcinoma progression via activating Wnt/beta-catenin signaling. Mol Oncol.

[13] He Y, Xu Y, Yu X, Sun Z, Guo W (2021). The vital roles of LINC00662 in human cancers. Front Cell Dev Biol.

[14] Cersosimo F, Lonardi S, Bernardini G, Telfer B, Mandelli GE, Santucci A, Vermi W, Giurisato E (2020). Tumor-associated macrophages in osteosarcoma: from mechanisms to therapy. Int J Mol Sci.

[15] Condeelis J, Pollard JW (2006). Macrophages: obligate partners for tumor cell migration, invasion, and metastasis. Cell.

[16] Han Y, Guo W, Ren T, Huang Y, Wang S, Liu K, Zheng B, Yang K, Zhang H, Liang X (2019). Tumor-associated macrophages promote lung metastasis and induce epithelial-mesenchymal transition in osteosarcoma by activating the COX-2/STAT3 axis. Cancer Lett.

[17] Dumars C, Ngyuen JM, Gaultier A, Lanel R, Corradini N, Gouin F, Heymann D, Heymann MF (2016). Dysregulation of macrophage polarization is associated with the metastatic process in osteosarcoma. Oncotarget.

[18] Segaliny AI, Mohamadi A, Dizier B, Lokajczyk A, Brion R, Lanel R, Amiaud J, Charrier C, Boisson-Vidal C, Heymann D (2015). Interleukin-34 promotes tumor progression and metastatic process in osteosarcoma through induction of angiogenesis and macrophage recruitment. Int J Cancer.

[19] Zhang Y, Lu W, Chen Y, Lin Y, Yang X, Wang H, Liu Z (2021). The miR-19b-3p-MAP2K3-STAT3 feedback loop regulates cell proliferation and invasion in esophageal squamous cell carcinoma. Mol Oncol.

[20] Zhang Y, Liu Z, Yang X, Lu W, Chen Y, Lin Y, Wang J, Lin S, Yun JP (2021). H3K27 acetylation activated-COL6A1 promotes osteosarcoma lung metastasis by repressing STAT1 and activating pulmonary cancer-associated fibroblasts. Theranostics.

[21] Huang J, Lin F, Xu C, Xu Y (2021). LINC00662 facilitates osteosarcoma progression via sponging miR-103a-3p and regulating SIK2 expression. J Tissue Eng Regen Med.

[22] Yu M, Lu W, Cao Z, Xuan T (2021). LncRNA LINC00662 exerts an oncogenic effect on osteosarcoma by the miR-16-5p/ITPR1 Axis. J Oncol.

[23] Ji W, Jiao J, Cheng C, Xiao Y, Shao J, Liu H (2021). A positive feedback loop of LINC00662 and STAT3 promotes malignant phenotype of glioma. Pathol Res Pract.

[24] Mohapatra S, Pioppini C, Ozpolat B, Calin GA (2021). Non-coding RNAs regulation of macrophage polarization in cancer. Mol Cancer.

[25] Arabpour M, Saghazadeh A, Rezaei N (2021). Anti-inflammatory and M2 macrophage polarization-promoting effect of mesenchymal stem cell-derived exosomes. Int Immunopharmacol.

[26] Atri C, Guerfali FZ, Laouini D (2018). Role of human macrophage polarization in inflammation during infectious diseases. Int J Mol Sci.

[27] Zhao X, Wu Q, Gong X, Liu J, Ma Y (2021). Osteosarcoma: a review of current and future therapeutic approaches. Biomed Eng Online.

[28] Tai WT, Chen YL, Chu PY, Chen LJ, Hung MH, Shiau CW, Huang JW, Tsai MH, Chen KF (2016). Protein tyrosine phosphatase 1B dephosphorylates PITX1 and regulates p120RasGAP in hepatocellular carcinoma. Hepatology.

[29] Lord RV, Brabender J, Wickramasinghe K, DeMeester SR, Holscher A, Schneider PM, Danenberg PV, DeMeester TR (2005). Increased CDX2 and decreased PITX1 homeobox gene expression in Barrett's esophagus and Barrett's-associated adenocarcinoma. Surgery.

[30] Ohira T, Nakagawa S, Takeshita J, Aburatani H, Kugoh H (2021). PITX1 inhibits the growth and proliferation of melanoma cells through regulation of SOX family genes. Sci Rep.

[31] Zhong C, Zhang Q, Zhang M, Qi Y, Duan S (2021). LINC00662: a new oncogenic lncRNA with great potential. J Cell Physiol.

[32] Fan H, Liu G, Zhao C, Li X, Yang X (2017). Transcription factor Oct4 promotes osteosarcoma by regulating lncRNA AK055347. Oncol Lett.

[33] Bosch-Barrera J, Queralt B, Menendez JA (2017). Targeting STAT3 with silibinin to improve cancer therapeutics. Cancer Treat Rev.

[34] Liu Y, Liao S, Bennett S, Tang H, Song D, Wood D, Zhan X, Xu J (2021). STAT3 and its targeting inhibitors in osteosarcoma. Cell Prolif.

[35] Huang Q, Zhong Y, Dong H, Zheng Q, Shi S, Zhu K, Qu X, Hu W, Zhang X, Wang Y (2020). Revisiting signal transducer and activator of transcription 3 (STAT3) as an anticancer target and its inhibitor discovery: where are we and where should we go?. Eur J Med Chem.

[36] Vitale I, Manic G, Coussens LM, Kroemer G, Galluzzi L (2019). Macrophages and metabolism in the tumor microenvironment. Cell Metab.

[37] Noy R, Pollard JW (2014). Tumor-associated macrophages: from mechanisms to therapy. Immunity.

[38] Rohrle N, Knott MML, Anz D (2020). CCL22 signaling in the tumor environment. Adv Exp Med Biol.

[39] Wei C, Yang C, Wang S, Shi D, Zhang C, Lin X, Xiong B (2019). M2 macrophages confer resistance to 5-fluorouracil in colorectal cancer through the activation of CCL22/PI3K/AKT signaling. Onco Targets Ther.

[40] Wang D, Yang L, Yue D, Cao L, Li L, Wang D, Ping Y, Shen Z, Zheng Y, Wang L (2019). Macrophage-derived CCL22 promotes an immunosuppressive tumor microenvironment via IL-8 in malignant pleural effusion. Cancer Lett.

