# Correction to: PITX1 suppresses osteosarcoma metastasis through exosomal LINC00662-mediated M2 macrophage polarization

**DOI:** 10.1007/s10585-023-10247-1

**Published:** 2023-12-08

**Authors:** Ying Zhang, Yelong Chen, Chuangzhen Chen, Huancheng Guo, Chunbin Zhou, Hu Wang, Zhaoyong Liu

**Affiliations:** 1 Department of Radiotherapy, Cancer Hospital of Shantou University Medical College, No. 7 Raoping Road, Shantou, 515041 Guangdong China; 2 Department of Orthopaedics, First Affiliated Hospital of Shantou University Medical College, No.57 Changping Road, Shantou, 515041 Guangdong China

## Correction to: Clinical & Experimental Metastasis (2023) 40:79–93 10.1007/s10585-022-10192-5

The original version of this article unfortunately contained some incorrect representative images. The wound healing images of MG63 cells in Fig. 1D had been misused with other cell line during figure assembly. The correct image of the Fig. 1 appears below.

**Fig. 1:**
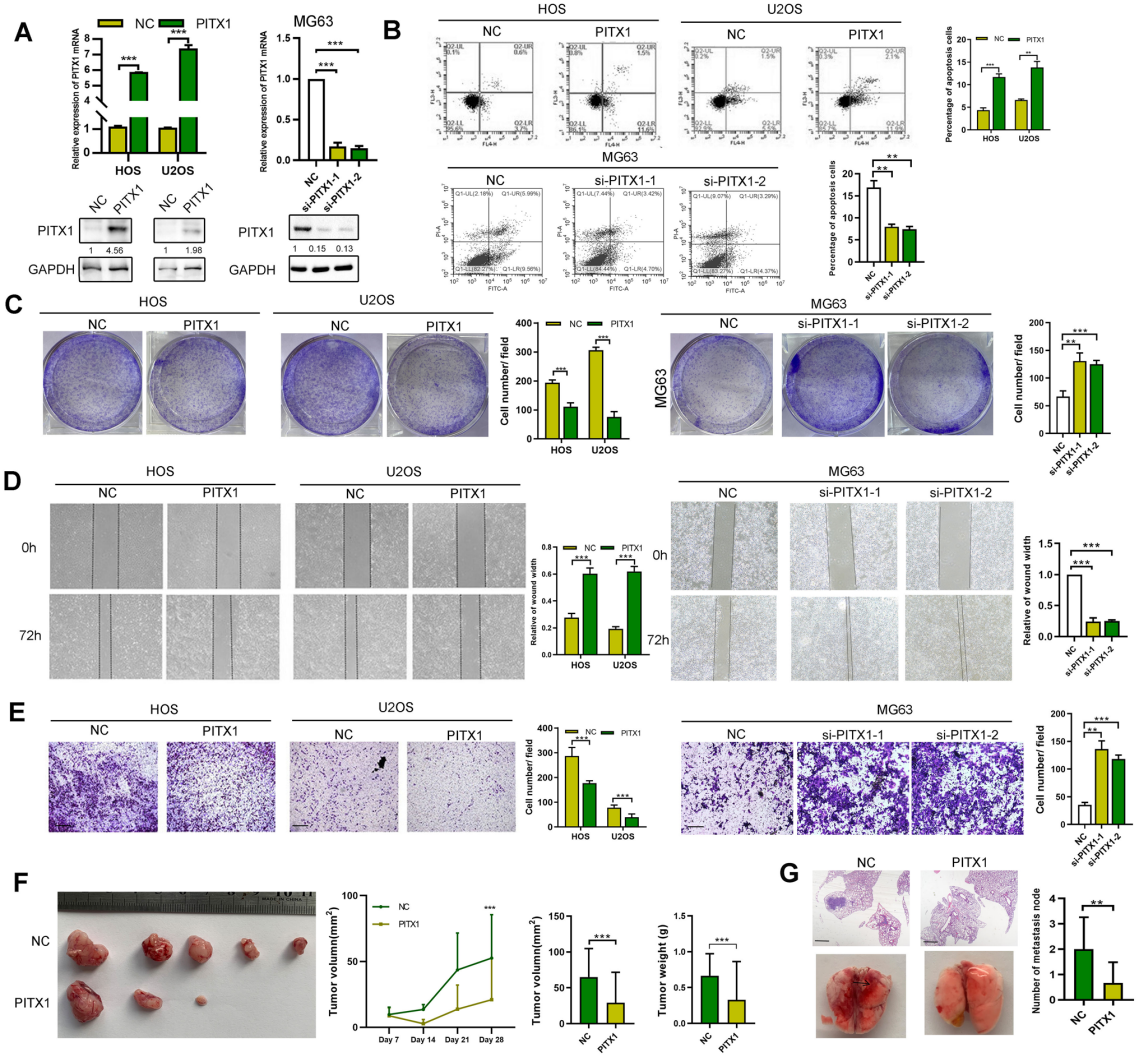
Corrected figure for original Fig. 1D. Wound healing assay was performed to measure the migration ability of control (NC) and PITX1-knockdown OS cells

The authors confirm that the corrections made in this erratum do not affect the original conclusions.

The original article has been corrected.

