# Supplementary figures and images for: PITX1 suppresses osteosarcoma metastasis through exosomal LINC00662-mediated M2 macrophage polarization
Source: Clin Exp Metastasis. 2022 Nov 5;40(1):79–93. doi: 10.1007/s10585-022-10192-5 (PMC9898340; doi:10.1007/s10585-022-10192-5)

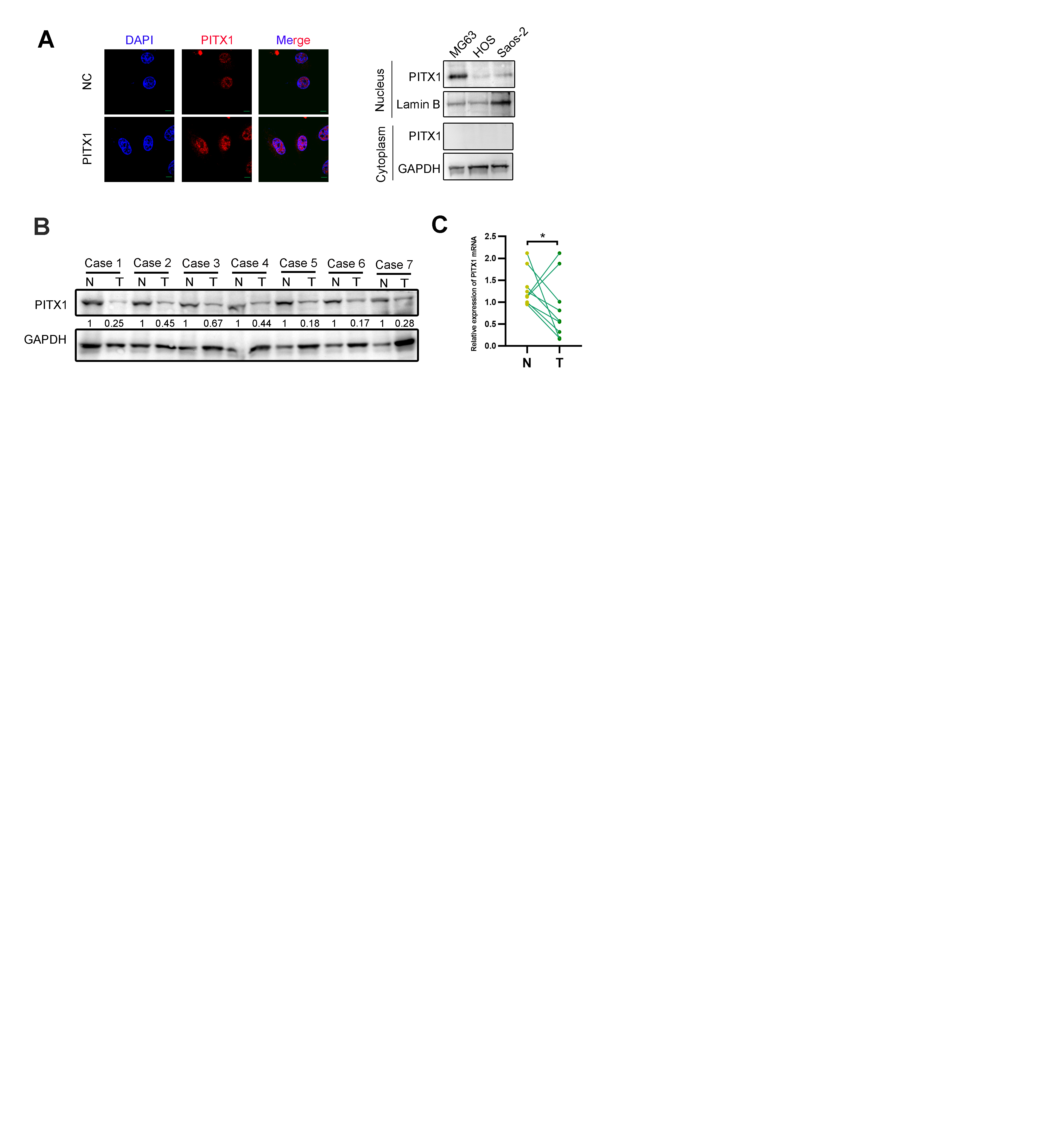

Supplement: Supplementary file 1 — Supplementary file1 (TIF 64397 kb)—Fig. 1 A Immunofluorescence and western blotting showing PITX1 was mainly located in the nucleus (Scale bars: 20 μm). B Expression of PITX1 in OS and normal tissues was detected by western blotting (n=7 pairs). C Expression of PITX1 in OS and normal tissues was detected by qRT-PCR (n=9) [file 10585_2022_10192_MOESM1_ESM.tif]

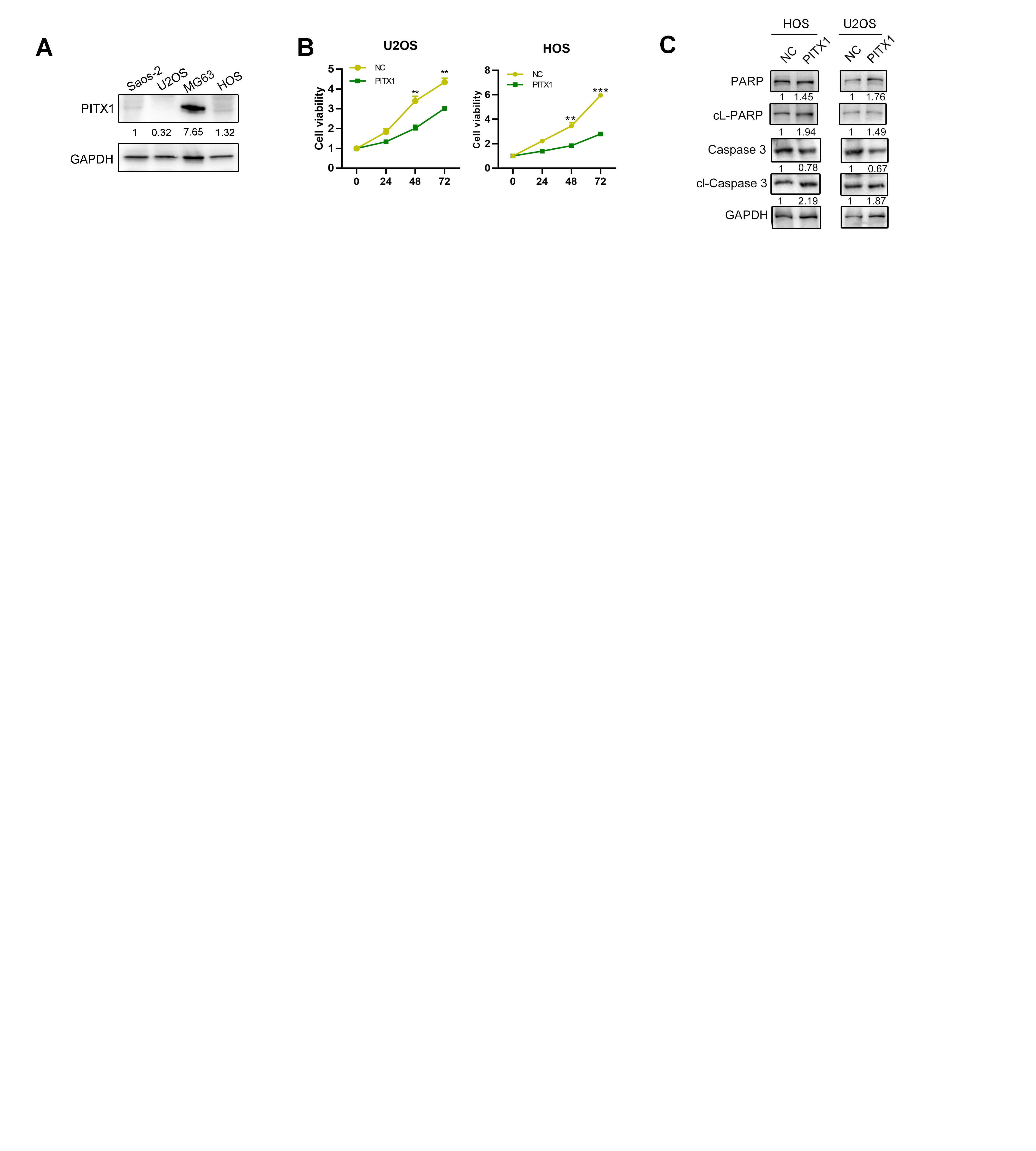

Supplement: Supplementary file 2 — Supplementary file2 (TIF 61306 kb)—Fig. 2 A Expression of PITX1 was detected by western blotting in OS cells. B Cell proliferation of PITX1-overexpressing or NC cells was performed using a CCK8 assay. C Expression of apoptosis biomarkers of PITX1 or NC cells was detected using western blotting [file 10585_2022_10192_MOESM2_ESM.tif]

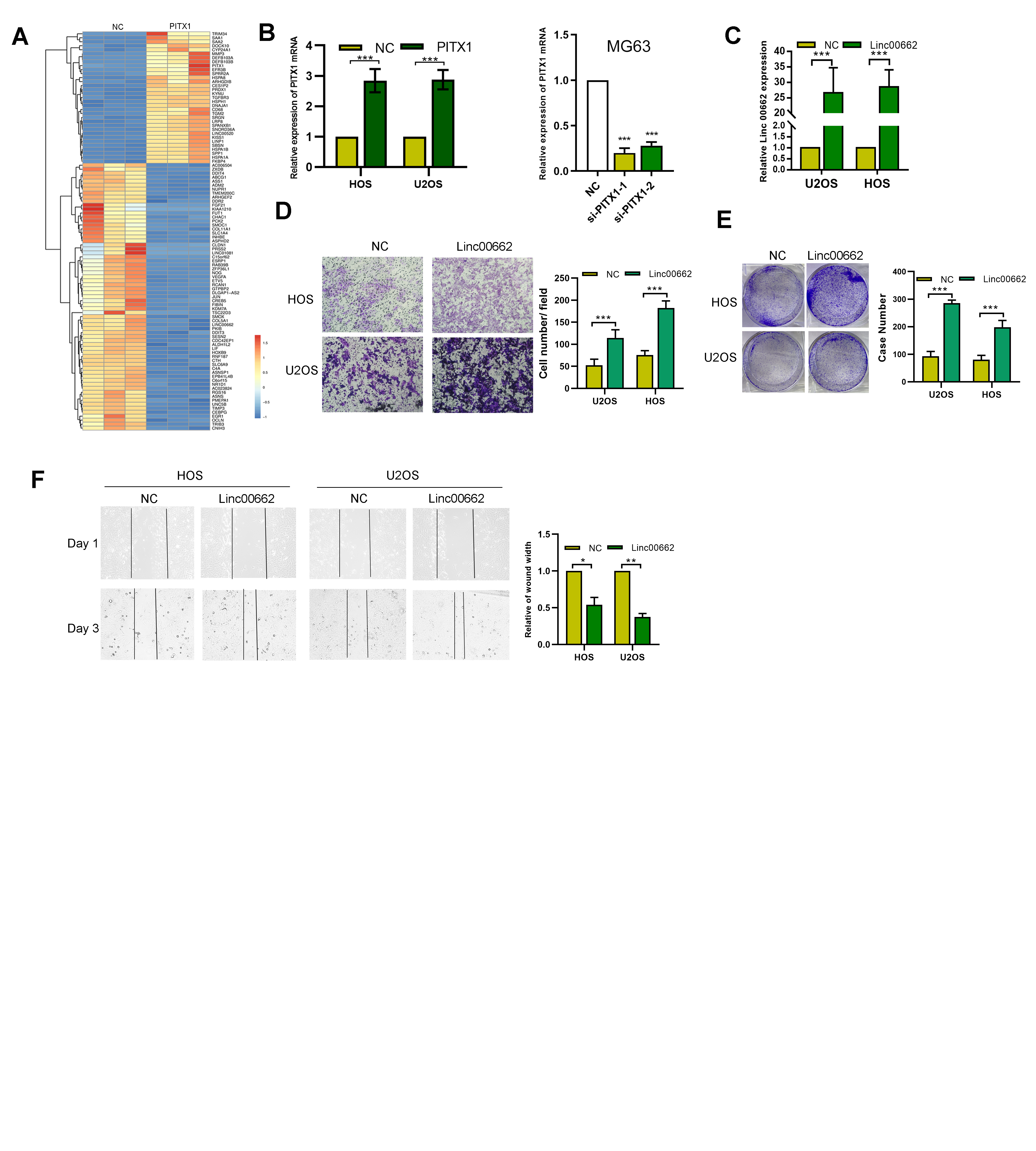

Supplement: Supplementary file 3 — Supplementary file3 (TIF 12084 kb)—Fig. 3 A Differentially-expressed genes of NC and PITX1-transfected cells are shown in a heatmap. B Transfection efficiency was confirmed by qRT-PCR showing expression of PITX1 in NC and PITX1-overexpressing or -knockdown OS cell lines. C Expression of LINC00662 in NC or LINC00662 stably-expressing HOS and U2OS cells. D Transwell assay of NC or LINC00662 stably-expressing HOS and U2OS cells. E Colony formation of NC or LINC00662 stably-expressing HOS and U2OS cells. F Wound healing assay performed on NC or LINC00662 stably-expressing HOS and U2OS cells [file 10585_2022_10192_MOESM3_ESM.tif]

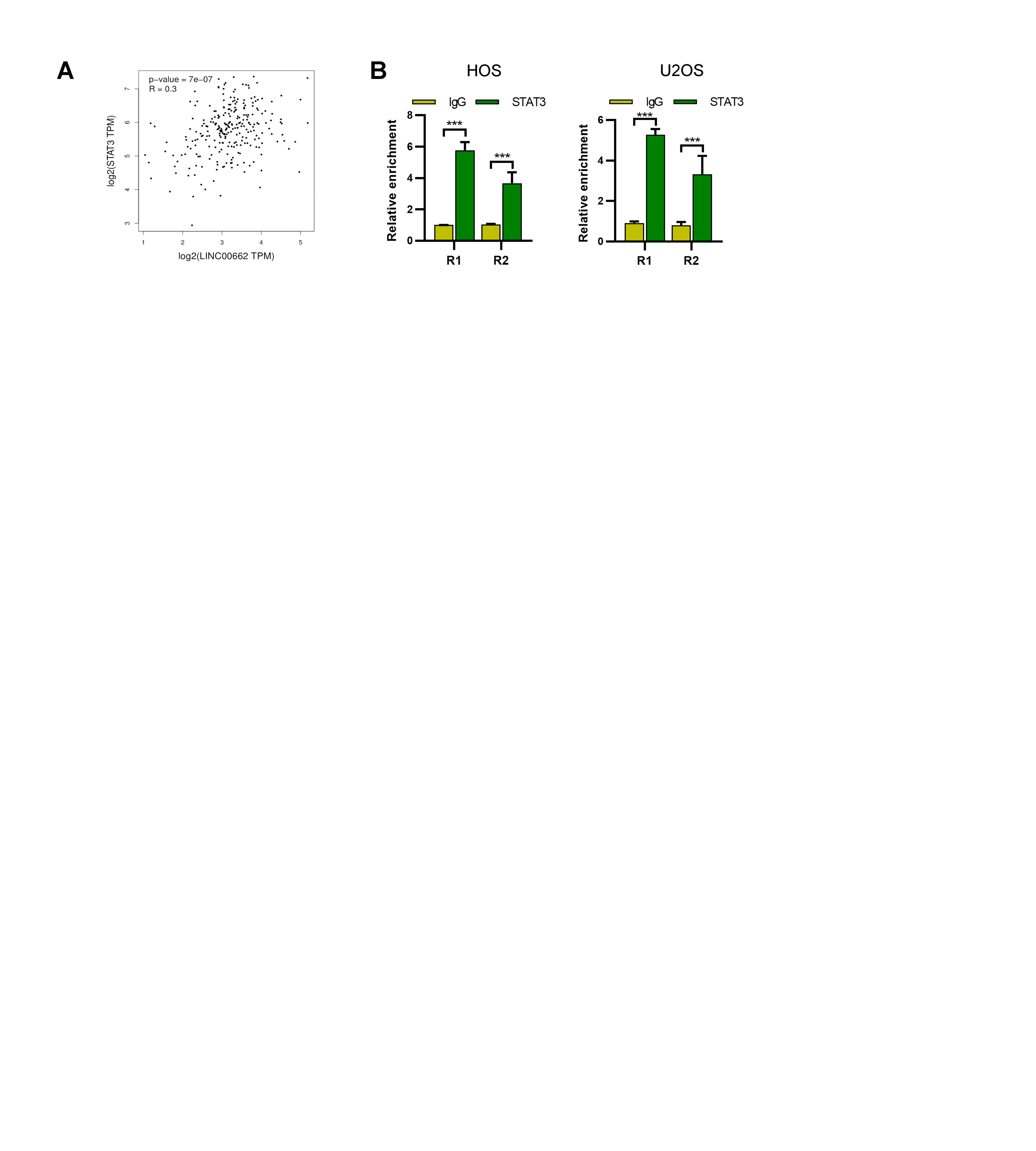

Supplement: Supplementary file 4 — Supplementary file4 (TIF 60292 kb)—Fig. 4 A Correlation of STAT3 and LINC00662 expression in TCGA sarcoma cases. B ChIP assay was used to determine the binding of STAT3 at the LINC00662 promoter at the predicted sites (R1 and R2) [file 10585_2022_10192_MOESM4_ESM.tif]

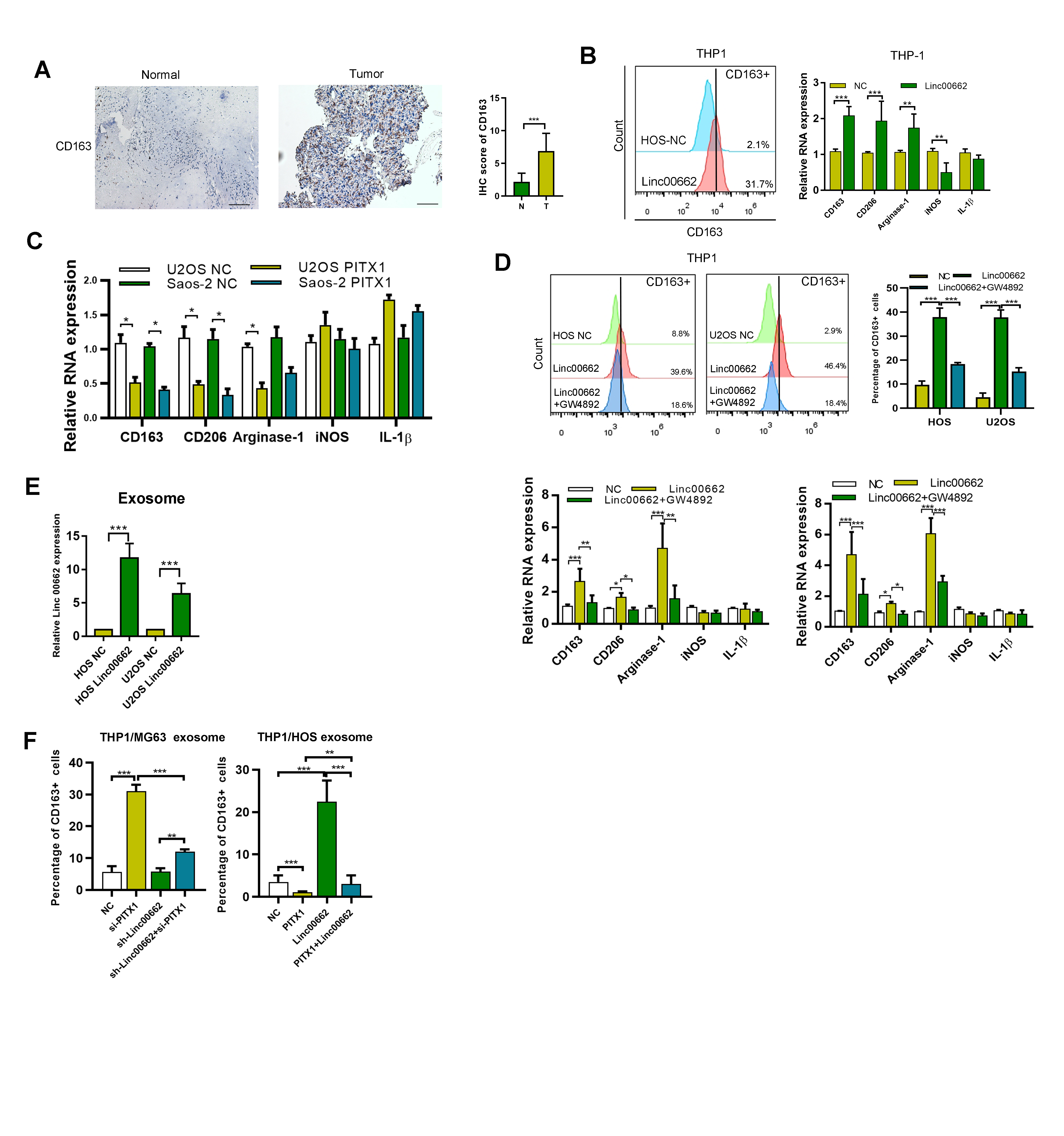

Supplement: Supplementary file 5 — Supplementary file5 (TIF 8920 kb)—Fig. 5 A Expression of CD163 in normal (n=5) and OS tissues (n=45; Scale bars: 100 μm). B PMA-treated THP-1 cells were co-cultured with HOS cells transfected with a control or LINC00662 expression vector. Expression of CD163 was determined using flow cytometry and expression typical M2 markers were detected by qPCR. C PMA-treated THP-1 cells were co-cultured with NC, PITX1-overexpressing OS cells, the expression of M2 macrophage biomarkers (CD163, CD206 and arginase-1) and M1 macrophage (iNOS and IL-1β) were determined qRT-PCR. D PMA-treated THP-1 cells were co-cultured with NC or LINC00662-overexpressing or GW4892-treated OS cells. Expression of CD163 was determined using flow cytometry and expression typical M2 markers were detected by qPCR. E Expression of LINC00662 in PMA-treated THP-1 cells co-cultured with LINC00662-overexpressing or control OS cells. F PMA-treated THP-1 cells were co-cultured with exosomes derived from NC or PITX1-overexpressing or -knockdown OS cells, or with LINC00662-overexpressing or -knockdown OS cells. Expression of CD163 was determined using flow cytometry [file 10585_2022_10192_MOESM5_ESM.tif]
