# Supplementary material for: PITX1 suppresses osteosarcoma metastasis through exosomal LINC00662-mediated M2 macrophage polarization
Source: Clin Exp Metastasis. 2022 Nov 5;40(1):79–93. doi: 10.1007/s10585-022-10192-5 (PMC9898340; doi:10.1007/s10585-022-10192-5)
Supplement: Supplementary file 6 — Supplementary file6 (DOCX 16 kb) [file 10585_2022_10192_MOESM6_ESM.docx]

| **Gene** | **F** | **R** |
| --- | --- | --- |
| **CD68** | GGAAATGCCACGGTTCATCCA | TGGGGTTCAGTACAGAGATGC |
| **CD206** | GGGTTGCTATCACTCTCTATGC | TTTCTTGTCTGTTGCCGTAGTT |
| **Arginase** | GTGGAAACTTGCATGGACAAC | AATCCTGGCACATCGGGAATC |
| **IL-10** | CCTCCGTCTGTGTGGTTTGAA | CACTGCGGTAAGGTCATAGGA |
| **iNOS** | GTTCCAGATGAATACTGGCAGTC | GCAACTGAACACTATCTTTCCCT |
| **CD163** | TTTGGACAAGCCGTGACTAGA | CATTCCCGGTGTTGACATTCC |
| **IL-1β** | ATGATGGCTTATTACAGTGGCAA | GTCGGAGATTCGTAGCTGGA |
| **Vimentin** | GCCCTAGACGAACTGGGTC | GGCTGCAACTGCCTAATGAG |
| **N-cadherin** | GCCCCTCAAGTGTTACCTCAA | AGCCGAGTGATGGTCCAATTT |
| **E-cadherin** | CGAGAGCTACACGTTCACGG | GGGTGTCGAGGGAAAAATAGG |
| **Linc00662** | GTCCTGCAGGCGTACAACTA | CTTCTCGCTAGCAGGTGAGG |
| **IL-13** | CCTCATGGCGCTTTTGTTGAC | TCTGGTTCTGGGTGATGTTGA |
| **CCL1** | CTCATTTGCGGAGCAAGAGAT | GCCTCTGAACCCATCCAACTG |
| **CCL5** | CCAGCAGTCGTCTTTGTCAC | CTCTGGGTTGGCACACACTT |
| **CCL13** | CTCAACGTCCCATCTACTTGC | TCTTCAGGGTGTGAGCTTTCC |
| **CCL14** | CCAAGCCCGGAATTGTCTTCA | GGGTTGGTACAGACGGAATGG |
| **CCL17** | CGGACCCCAACAACAAGAGA | ACTGTGGCTCTTCTTCGTCC |
| **CCL18** | CTCTGCTGCCTCGTCTATACCT | CTTGGTTAGGAGGATGACACCT |
| **CCL22** | GATCTGTGCCGATCCCAGAG | AGGGAATGCAGAGAGTTGGC |
| **CCL23** | CATCTCCTACACCCCACGAAG | GGGTTGGCACAGAAACGTC |
| **CCL24** | ACATCATCCCTACGGGCTCT | CTTGGGGTCGCCACAGAAC |
| **CXCL10** | GTGGCATTCAAGGAGTACCTC | TGATGGCCTTCGATTCTGGATT |
| **IL-1R** | TCTGTTCTTGGGAATCCATGG | TCAGTGATGTTAACTGCCTCCAG |
| **IL-8** | AAACCACCGGAAGGAACCAT | CCTTCACACAGAGCTGCAGAAA |
| **PITX1** | CTAGAGGCCACGTTCCAGAG | TGGTTACGCTCGCGCTTAC |
| **Primer 1** | TCCACACAAATCAGGGCAGT | CCCTAATTTCCAGGCAGTGT |
| **Primer 2** | TCCCTCATGCTCAAAATTCACA | CCAGTTCATCCGCTTTTGGG |
| **GAPDH** | GAAGGTGAAGGTCGGAGTCAACG | TGCCATGGGTGGAATCATATTGG |

**Supplemental Table 1. Primers**
